# The hypoglycemic metabolites and potential mechanisms of *Lilium lancifolium* (*Juandan* lily)

**DOI:** 10.3389/fphar.2026.1806579

**Published:** 2026-06-08

**Authors:** Qinxuan Wu, Kunyu Xiao, Di Zhao, Rui Tang, Weiping Liu, Feng Li, Hexiang Wang, Fengming Chen

**Affiliations:** 1 Hunan Provincial Key Laboratory of the Traditional Chinese Medicine Agricultural Biogenomics, Changsha Medical University, Changsha, China; 2 Hunan Provincial Key Laboratory of the Research and Development of Novel Pharmaceutical Preparations, Changsha Medical University, Changsha, China; 3 Yuelushan Laboratory, Changsha, China

**Keywords:** active metabolites, hypoglycemic activity, Lilium lancifolium thunb., molecular docking, regaloside B, UPLC-Q-TOF-MS/MS

## Abstract

In Chinese folk medicine, lily bulbs have traditionally been used to help lower blood glucose levels. Numerous studies have demonstrated that extracts from lily bulbs possess significant hypoglycemic activity. However, the specific active metabolites and their underlying mechanisms remain unclear. This study first evaluated the hypoglycemic activities of extracts from four common lily species: *Lilium lancifolium* Thunb (*Juandan* lily), *Lilium davidii* var. Unicolor (*Lanzhou* lily), *Lilium brownii* var. Viridulum (*Longya* lily), and *Guiyanghong* lily. The findings revealed that *Juandan* lily extract exhibited the most potent hypoglycemic activity. Subsequently, UPLC-Q-TOF-MS analysis was employed to profile the major metabolites of *Juandan* lily, resulting in the identification of 25 high-abundance metabolites. These included 8 regalosides, 5 flavonoids, 4 dioscin-type saponins, 5 phenylpropanoids, and 3 other types of metabolites. Notably, six of them were reported for the first time in lilies. To further investigate the hypoglycemic metabolites, three high-content metabolites (caffeic acid, regaloside A, and regaloside B) from *Juandan* lily were subjected to hypoglycemic activity assessment. The results indicated that both regaloside A and regaloside B possessed significant hypoglycemic activity, with regaloside B demonstrating the strongest effect, reducing blood glucose levels in hyperglycemic model mice from an initial values of 27.87 ± 0.67 mmol/L to 14.11 ± 1.09 mmol/L. Furthermore, regaloside A and B were found to inhibit postprandial blood glucose elevation in mice, with an efficacy comparable to that of the positive control drug. This effect was attributed to the inhibition of α-glucosidase activity. Molecular docking studies revealed that regaloside A and B could bind to α-glucosidase (including maltase-glucoamylase and sucrase-isomaltase). These interactions may inhibit the α-glucosidase activity and contribute to the observed hypoglycemic effect. This study elucidates the active metabolites and the potential mechanism underlying the hypoglycemic activity of lily bulbs for the first time, thereby providing a foundation for the development of novel hypoglycemic agents and the high-value utilization of lily resources.

## 1 Introduction

Lilii bulbus, derived from the dried bulbs of *Lilium lancifolium* Thunb., *Lilium brownii* var. Viridulum, or *Lilium pumilum* DC. in the Liliaceae family, is traditionally used in Chinese medicine for its effects of nourishing yin, moistening the lungs, calming the mind, and relieving restlessness (Xu, et al., 2024; Liang, et al., 2025). It is commonly used to treat conditions such as dry cough due to yin deficiency and palpitations caused by mental unrest. In China, lily bulbs are widely consumed as a medicinal food homologous substance (Guo, et al., 2024). Modern research has demonstrated that lily bulbs possess various biological activities, including antidepressant, hypoglycemic, sleep-improving, antioxidant, and immune-enhancing effects (Qin, et al., 2022; Long et al., 2023). Chemical composition analyses indicate that lily bulbs are rich in bioactive metabolites such as polysaccharides, steroidal saponins, alkaloids, and phenolic compounds, which constitute the material basis for their pharmacological actions (Esposito, et al., 2013; Munafo et al., 2015; Wang, et al., 2019). In recent years, the hypoglycemic effect of lily bulbs has become a research focus. Studies have shown that lily polysaccharide extracts exhibit significant hypoglycemic activity by repairing pancreatic β-cells, promoting insulin secretion, and enhancing peripheral tissue sensitivity to insulin (Zhang, et al., 2025). Additionally, steroidal saponin extracts from lily bulbs competitively inhibit α-glucosidase activity in the small intestinal mucosa, thereby delaying carbohydrate digestion and glucose absorption to achieve hypoglycemic effects (Zhu, et al., 2013). Despite numerous studies confirming the significant hypoglycemic activity of lily bulb extracts, the specific active metabolites responsible for this effect remain unclear and warrant further investigation.

Type 2 diabetes mellitus (T2DM) is a chronic metabolic disorder characterized primarily by insulin resistance and relative insulin deficiency. Its main clinical feature is chronic hyperglycemia, often accompanied by obesity, dyslipidemia, and hypertension. Long-term T2DM can lead to severe complications affecting the cardiovascular and cerebrovascular systems, kidneys, retina, and nerves (Kalyani, et al., 2025; Grover, et al., 2025). Currently, the global prevalence of T2DM continues to rise. In 2024, the worldwide diabetes prevalence rate reached 11.11%, with the total number of patients exceeding 580 million. It is projected that by 2050, the global number of diabetes patients will soar to 850 million. China has the highest number of diabetes patients globally, making T2DM a significant public health challenge for human society (Zhu, et al., 2013; Chen, et al., 2025). Modern medical treatment of T2DM primarily relies on lifestyle interventions and stepwise hypoglycemic drug therapy. Commonly used medications include first-line foundational drugs such as metformin, insulin secretagogues like sulfonylureas and glinides, insulin sensitizers such as thiazolidinediones, and α-glucosidase inhibitors. These drugs demonstrate clear hypoglycemic effects with rapid onset and are highly effective in controlling blood glucose and preventing acute complications. However, they also exhibit notable drawbacks, such as significant long-term adverse effects on the liver and kidneys, suboptimal hypoglycemic efficacy owing to single-target mechanisms, and the risk of inducing hypoglycemia with potent glucose-lowering agents (Zhu, et al., 2013; Attri, et al., 2024). In contrast, traditional Chinese herbal medicines or natural products hold unique advantages in the treatment or prevention of T2DM due to their lower toxicity, fewer side effects, and multi-target actions. Consequently, they have emerged as a new research hotspot in the field of hypoglycemic therapy.

α-Glucosidase, which mainly include maltase-glucoamylase and sucrase-isomaltase, is a key digestive enzyme located on the brush border membrane of the small intestine. Its primary function is to catalyze the hydrolysis of non-reducing terminal α-1.4 glycosidic bonds in carbohydrates (such as starch and sucrose), releasing α-glucose for absorption and utilization by the human body (Liao et al., 2025). By competitively inhibiting α-glucosidase activity, these agents delay the breakdown of carbohydrates into monosaccharides and slow glucose absorption, thereby lowering blood glucose levels (Syam et al., 2021; Zhou et al., 2022). Lily bulb extracts and their active metabolites exhibit significant hypoglycemic activity; however, whether their mechanisms involve this core pathway remains to be further investigated.

This study compared the hypoglycemic activities of extracts from four different lily varieties and found that the extract of *Juandan lily* exhibited significant hypoglycemic activity. Subsequently, the major metabolites of *Juandan lily* were identified using ultra performance liquid chromatography quadrupole time-of-flight mass spectrometry (UPLC-Q-TOF-MS), and the hypoglycemic activities and mechanisms of its primary constituents (caffeic acid, regaloside A, and regaloside B) were investigated. This study is the first time to elucidate the active hypoglycemic metabolites of lily bulbs and their corresponding mechanisms, laying a foundation for the development of hypoglycemic drugs and the high-value utilization of lily resources.

## 2 Materials and methods

### 2.1 Instruments and reagents

An Agilent 1,290 series ultra-high performance liquid chromatography (UPLC) system coupled with a 6,540 quadrupole time-of-flight mass spectrometer (Q-TOF-MS; Agilent Technologies, United States of America) was used. Ultra-pure water was prepared using a Milli-Q Advantage A10 system (Millipore, United States of America). Blood glucose levels were measured using a glucose meter and corresponding test strips (OneTouch UltraEasy; Johnson & Johnson Medical Devices, China). Caffeic acid (≥98%), regaloside A (≥98%), and regaloside B (≥98%) (Shanghai Standard Biotech Co., Ltd., China) were used as reference standards to investigate the hypoglycemic active metabolites in lily bulbs. Streptozotocin (≥99%, Dalian Meilun Biotechnology Co., Ltd.) was used to establish hyperglycemic mouse models. Acarbose (≥95%, Shanghai Yuanye Bio-Technology Co., Ltd.) served as the positive control. The high-fat diet used in this study was purchased from Jiangsu Synergy Pharmaceutical Bioengineering Co., Ltd. (Jiangsu, China) with type of XTHF45. The composition of the diet includes 23.3% lard, 2.9% soybean oil, 46.3% carbohydrates, and 23.6% protein. Acetonitrile and formic acid (Sinopharm Chemical Reagent Co., Ltd.) were used for UPLC-Q-TOF-MS analyses.

### 2.2 Pre-treatment of samples

Fresh bulbs of *L. lancifolium* (*Juandan* lily), *L. davidii* (*Lanzhou* lily), *L. longiflorum* (*Longya* lily), and *Guiyanghong* lily were collected from the Medicinal Plant Garden of Changsha Medical University (Hunan, China, GPS coordinates are E112◦87′40.15″ and N28◦29′64.27″). The different lily varieties were identified by Professor Qinxuan Wu of Changsha Medical University. Voucher specimens have been deposited at the Herbarium of Changsha Medical University with the accession numbers (*Juandan* lily-20250912, *Lanzhou* lily-20250,912, *Longya* lily-20250912, and *Longyahong* lily-20250912). Approximately 10 kg of bulbs were collected for each variety, cleaned of soil, and dried in an oven at 80 °C. After drying, the bulbs were ground into powder. The dried powder was then subjected to reflux extraction with 30 L of 80% ethanol twice. The extracts were combined, and the solvent was evaporated to obtain the extracts. The extracts were freeze-dried and stored for further use. One portion was used for UPLC-Q-TOF-MS analysis, and another portion was used for *in vivo* and *in vitro* hypoglycemic activity studies.

### 2.3 Preparation of lily extracts and standard solutions

Exactly 5.00 mg of dried lily extracts from different varieties and reference standards (caffeic acid, regaloside A, and regaloside B) were accurately weighed and dissolved in 5 mL of methanol under sonication. The solutions were then filtered through a 0.22 µm microporous membrane. A portion of the filtered solution was transferred into UPLC vials for the identification of major metabolites in the lily samples.

### 2.4 UPLC-Q-TOF-MS conditions for identifying metabolites

The major metabolites in lily samples were analyzed using UPLC (1,290)-Q-TOF-MS (6,540) technology. An Agilent C18 column (100 mm × 2.1 mm, 1.7 µm) was employed as the stationary phase. The mobile phase consisted of 0.1% formic acid in water (A) and in acetonitrile (B), with gradient elution as follows: 0–5 min, 5%–30% B; 5–25 min, 30%–70% B; 25–35 min, 70%–90% B. The detection wavelength was set at 254 nm. The flow rate was 0.3 mL/min, and the injection volume was 5 µL. An ESI ion source was used in negative ion mode. The drying gas flow rate was 11 L/min at 350 °C, the sheath gas flow rate was 10 L/min at 350 °C, and the nebulizer gas pressure was 55 psig. The capillary voltage was set to 3500 V, and the nozzle voltage was 100 V. High-resolution tandem mass spectrometry (MS/MS) data were acquired in targeted MS/MS mode with collision energies rang from 10 to 60 eV. The TOF-MS was continuously calibrated using a reference solution (*m/z* 112.9855 and 966.0007) to ensure high-accuracy mass measurements.

### 2.5 *In Vivo* and *in vitro* hypoglycemic activity study

#### 2.5.1 Evaluation of hypoglycemic activity of different lily extracts

Eighty Kunming mice (18–24 g) were selected and acclimatized for 1 week before being fed a high-fat diet for 30 days. Except for the normal control group (10 mice), the remaining 70 mice were intraperitoneally injected with streptozotocin (60 mg/kg), while the normal control group received an equal volume of distilled water. Injections were administered consecutively for 3 days. On the third day after injection, fasting blood glucose levels were measured. Mice with fasting blood glucose levels >11.1 mmol/L but <33.3 mmol/L were considered successful models (Zhou, et al., 2022). The successfully modeled mice were divided into seven groups based on body weight and fasting blood glucose levels: model control group (MC); positive control group (acarbose); *Juandan* lily extract group (JD); *Lanzhou* lily extract group (LZ); *Longya* lily extract group (LY); and Guiyanghong lily extract group (GYH), with 10 mice per group. The normal control and model groups were administered distilled water daily by gavage, while the positive control group received acarbose (100 mg/kg) daily. For comparison with the hypoglycemic activity of acarbose, the dose of the four lily bulb extracts was also designed as 100 mg/kg for 28 consecutive days. Fasting blood glucose levels were measured on days 0, 7, 14, 21, and 28. All experiments and procedures were conducted in accordance with the Regulations of Experimental Animal Administration issued by the State Committee of Science and Technology of China (20170103). This study was approved by the Biomedical Ethics Committee of Changsha Medical University (CMU-2024-0056).

#### 2.5.2 Evaluation of the hypoglycemic activity of caffeic acid, regaloside A, and regaloside B

Seventy Kunming mice (18–24 g) were selected. Except for the normal control group (10 mice), the remaining 60 mice were modeled using the same method as described in the evaluation of lily extracts. The successfully modeled mice were divided into six groups based on body weight and fasting blood glucose levels: model group (MC); positive control group (acarbose); caffeic acid group (C-A); regaloside A group (R-A); and regaloside B group (R-B), with 10 mice per group. The normal control and model groups were administered distilled water daily by gavage, while the positive control group received acarbose (100 mg/kg) daily. For comparison the hypoglycemic activity of the three metabolites with acarbose, the dose of the these metabolites was also designed as 100 mg/kg for 28 consecutive days. Fasting blood glucose levels were measured on days 0, 7, 14, 21, and 28.

#### 2.5.3 Evaluation of the postprandial blood glucose-lowering activity of lily extracts and three metabolites

First, the postprandial blood glucose-lowering activity of different lily extracts was evaluated. Sixty normal mice aged 4 weeks, weighing 18.0–24.0 g, were randomly divided into six groups based on body weight. All mice were received a sucrose solution at a dose of 2.0 g/kg. Except for the normal control group (NC), the remaining five groups were given acarbose (100 mg/kg), *Juandan* extract (100 mg/kg), *Lanzhou* lily extract (100 mg/kg), *Longya* lily extract (100 mg/kg), and *Guiyanghong* lily extract (100 mg/kg), respectively. Blood glucose levels were measured using a glucose meter at 0, 30, 60, 90, and 120 min. The evaluation of the postprandial blood glucose-lowering activity of caffeic acid, regaloside A, and regaloside B followed the same protocol as for the lily extracts, with each metabolite administered at a dose of 100 mg/kg per group.

### 2.6 *In Vitro* α-glucosidase inhibitory activity evaluation of lily extracts and three metabolites

The α-glucosidase inhibitory activity of four lily extracts and three metabolites was determined following the method described (Yang, et al., 2015) with slight modifications. Briefly, 120 μL of PBS (pH 6.8) and 20 μL of sample solutions at different concentrations were added to a 96-well plate, followed by 20 μL of α-glucosidase solution (0.24 U/mL). The mixture was gently mixed and incubated at 37 °C for 10 min to activate α-glucosidase. Then, 20 μL of 2.5 mmol/L pNPG solution was added as the substrate, mixed thoroughly, and further incubated at 37 °C for 10 min. The reaction was terminated by adding 80 μL of 0.2 mol/L anhydrous Na_2_CO_3_ solution, followed by mixing. The absorbance was measured at 405 nm. The inhibitory rate of lily extracts and the metabolites on α-glucosidase activity was calculated using the following formula. For acarbose, *Juandan* extract, and regaloside B, gradient dilutions were prepared, and the α-glucosidase inhibitory activity was measured using the same procedure. The IC_50_ values were calculated using GraphPad Prism 8 software.

$$\text{Inhibitory Rate }\left(\%\right)=\left[1\;‐\;\left(A_{1}\;‐\;A_{2}\right)\;/\;\left(A_{3}\;‐\;A_{4}\right)\right]\times 100\%$$

where: A_1_: Absorbance of the test sample; A_2_: Absorbance of the reaction system without α-glucosidase; A_3_: Absorbance of the reaction system without the test sample; A_4_: Absorbance of the reaction system without both the test sample and α-glucosidase.

### 2.7 Molecular docking of regaloside A and B with α-glucosidase (maltase-glucoamylase and sucrase-isomaltase)

To evaluate the interactions between regaloside A and B with α-glucosidase, molecular docking was performed to analyze their binding capabilities. The two-dimensional structures of regaloside A and regaloside B were retrieved from the PubChem database (https://pubchem.ncbi.nlm.nih.gov), and their SDF format files were downloaded. Three-dimensional structures of the ligands were constructed using Chem3D 20.0, and their spatial conformations were optimized through energy minimization to obtain stable initial structures. The optimized structures were then exported in PDB format. Hydrogen atoms were added and Gasteiger charges were calculated using AutoDockTools, and the structures were finally converted to PDBQT format for molecular docking.

The crystal structures of α-glucosidase-related target proteins were downloaded from the Protein Data Bank database (https://www.rcsb.org), including the N-terminal domain of maltase-glucoamylase (Nt-MGAM, PDB ID: 2QMJ), the C-terminal domain of maltase-glucoamylase (Ct-MGAM, PDB ID: 3TOP), and the N-terminal domain of sucrase-isomaltase (Nt-SI, PDB ID: 3LPP). Protein structures were preprocessed using PyMOL 3.1.1, including removal of water molecules, ligands, and heteroatoms. Subsequently, AutoDockTools was used to add polar hydrogens, assign Gasteiger charges, merge non-polar hydrogens, and save the structures in PDBQT format.

Molecular docking was performed using AutoDock Vina 1.1.2. The binding pocket regions were defined based on the positions of the co-crystallized ligands (acarbose in 2QMJ and 3TOP), and the geometric centers of these ligands were used as the center coordinates of the grid box. The grid box dimensions were set according to the spatial extent of the co-crystallized ligands to cover the active pocket and surrounding key amino acid residues. The docking results were visualized and analyzed using Discovery Studio 2019 to examine the types of interactions between the ligands and target proteins, including hydrogen bonding, hydrophobic interactions, π-π stacking, and electrostatic interactions. Additionally, the binding position and conformational characteristics of the ligands within the active pocket were analyzed to evaluate their potential modes of action.

### 2.8 Statistical analysis

Statistical analysis was conducted using IBM SPSS Statistics 25.0 and GraphPad Prism 9.0. Data are presented as mean ± SEM, and Levene’s test was used to assess normality and homogeneity of variances. When both normality and homogeneity of variances were satisfied (*p* > 0.05), statistical analysis was performed using ANOVA followed by LSD test. When normality or homogeneity of variances was not satisfied (*p* ≤ 0.05), the Kruskal–Wallis test was used. If the Kruskal–Wallis test showed statistical significance (*p* ≤ 0.05), Dunnett’s test was then applied for comparative analysis.

## 3 Results and discussion

### 3.1 The hypoglycemic activity of four lily extracts

**FIGURE 1:**
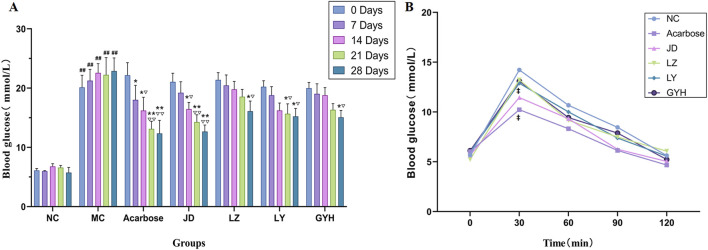
Study on the hypoglycemic activity of extracts from *Juandan* lily (JD), *Lanzhou* lily (LZ), *Longya* lily (LY), and *Guiyahong* lily (GYH) extracts. **(A)** Effects of four different lily extracts on blood glucose levels in hyperglycemic model mice (n = 8–10). **(B)** Effects of four different lily extracts on postprandial blood glucose (120 min) in normal mice (n = 10). ^##^ *p* < 0.01 compared the data with the normal control group (NC) in the same period; ^*^ *p* < 0.05, ^**^ *p* < 0.01 compared with the data at 0 days, ^▽^ *p* < 0.05, ^▽▽^ *p* < 0.01 compared the data with the model group in the same period; ^‡^ *p* < 0.05 compared with the data of NC at 30min; NC: normal control group; MC: model control group; JD: *Juandan* lily extract; LZ: *Lanzhou* lily extract; LY: *Longya* lily extract; GYH: *Guiyanghong* lily extract.

Numerous lily varieties exist, yet which possess hypoglycemic activity remains unknown. This study evaluated the hypoglycemic activity of extracts from four common lily species. As shown in Figure 1A, the blood glucose levels of the model group mice at day 0 (20.11 ± 2.06 mmol/L), day 7 (21.26 ± 1.89 mmol/L), day 14 (22.56 ± 1.56 mmol/L), day 21 (22.23 ± 2.90 mmol/L), and day 28 (22.87 ± 2.22 mmol/L) were significantly higher than those of the normal control group (*p* < 0.01), indicating the successful establishment of the hyperglycemic mice model induced by a high-fat diet combined with streptozotocin. The acarbose group exhibited significant hypoglycemic activity, with blood glucose levels decreasing from an initial values of 22.17 ± 2.12 mmol/L to 18.02 ± 2.45 (day 7, *p* < 0.05), 16.21 ± 2.23 (day 14, *p* < 0.05), 13.11 ± 1.28 mmol/L (day 21, *p* < 0.01), and 12.34 ± 2.21 mmol/L (day 28, *p* < 0.01). *Juandan* lily extract (JD) also demonstrated significant hypoglycemic activity, reducing blood glucose levels from an initial values of 21.05 ± 1.45 mmol/L (day 0) to 16.45 ± 1.13 (day 14, *p* < 0.05), 14.27 ± 1.23 mmol/L (day 21, *p* < 0.01), and 12.67 ± 1.09 mmol/L (day 28, *p* < 0.01). Compared to the model group, *Juandan* lily extract also significantly lowered blood glucose levels for the hyperglycemia mice (*p* < 0.05 or 0.01). Extracts from *Lanzhou* lily (LZ), *Longya* lily (LY), and *Longyahong* lily (LYH) also showed significant hypoglycemic effects, reducing blood glucose levels from initial values of 21.38 ± 1.23, 20.23 ± 1.01, and 19.98 ± 0.98 mmol/L (day 0) to 16.09 ± 1.72, 15.22 ± 1.36, and 15.06 ± 1.17 mmol/L (day 28, *p* < 0.05), respectively. However, the hypoglycemic activity of these three lily extracts was notably weaker than that of *Juandan* lily.

As shown in Figure 1B, acarbose significantly reduced postprandial blood glucose in mice, with a 28.1% decrease compared to the normal control group at 30 min postprandial (*p* < 0.05). *Juandan* lily extract also exhibited notable postprandial blood glucose-lowering activity, reducing levels by 19.5% compared to the normal control group at 30 min postprandial (*p* < 0.05). Extracts from *Lanzhou* lily, *Longya* lily, and *Longyahong* lily showed some postprandial blood glucose-lowering activity, though their effects were weaker than those of *Juandan* lily and acarbose. The above experimental results indicate that extracts from *Juandan* lily, Lanzhou lily, *Longya* lily, and *Longyahong* lily possess hypoglycemic activity, with *Juandan* lily extract demonstrating the strongest effect.

### 3.2 Identification of major metabolites in four lily extracts based on UPLC-Q-TOF-MS

**FIGURE 2:**
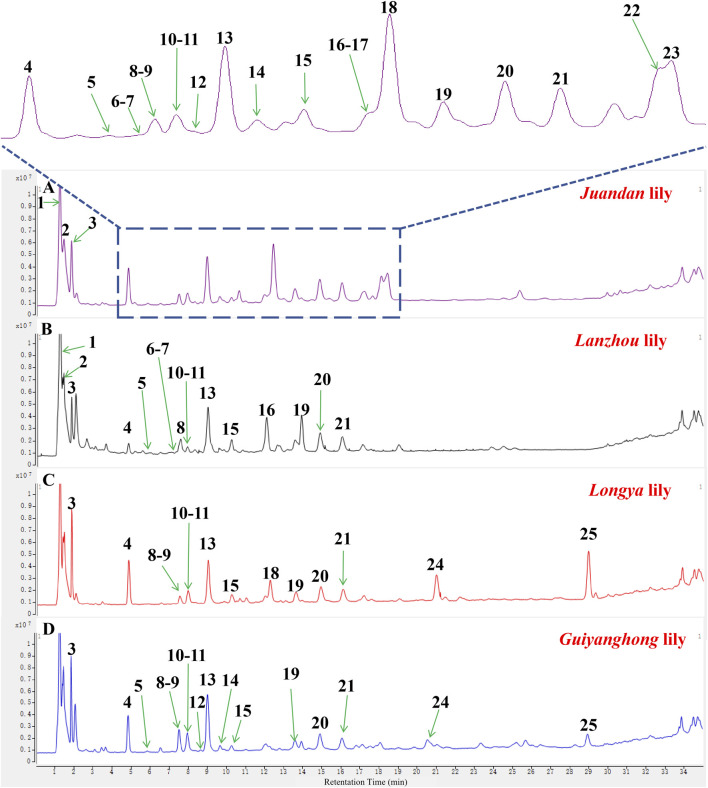
Identification of main metabolites in lilies by UPLC-Q-TOF-MS technology. Total ion chromatograms (TICs) and main metabolites (1–25) of *Juandan* lily **(A)**, *Lanzhou* lily **(B)**, *Longya* lily **(C)**, and *Guiyanghong* lily **(D)**.

*Juandan* lily extract demonstrated significant hypoglycemic activity. To elucidate its specific active constituents, we first identified the major metabolites in extracts from the four lily species using UPLC-Q-TOF-MS technology. A two-pronged strategy was employed for the identification of key metabolites (Liu, et al., 2020; Zhou, et al., 2022). Strategy one: First, a comprehensive database (Supplementary Table S1) of metabolites previously reported in lilies was compiled, containing information such as metabolite names, structural formulas, molecular formulas, and mass-to-charge ratios of 99 metabolites. In the total ion chromatograms (TICs, Figure 2) of the four lily extracts, metabolites corresponding to prominent mass spectrometry peaks (indicative of relatively high abundance) were selected. Their secondary mass spectra were acquired using targeted-MS/MS method. The primary and secondary mass spectra of these screened metabolites were then compared with the chemical information in Supplementary Table S1 for structural identification. Strategy two: For metabolites not previously reported in lilies and thus absent from Supplementary Table S1, structural identification was performed by applying established mass spectrometry fragmentation rules of flavonoids, regalosides, steroidal saponins, and polyphenols. Using these combined approaches, 25 relatively high-abundance chemical metabolites were identified in four lily. These include 8 regaloside, 5 flavonoids, 4 dioscin-type saponins, 5 phenylpropanoids, and 3 other types of metabolites. Notably, 6 of them are identified in lilies for the first time.

**FIGURE 3:**
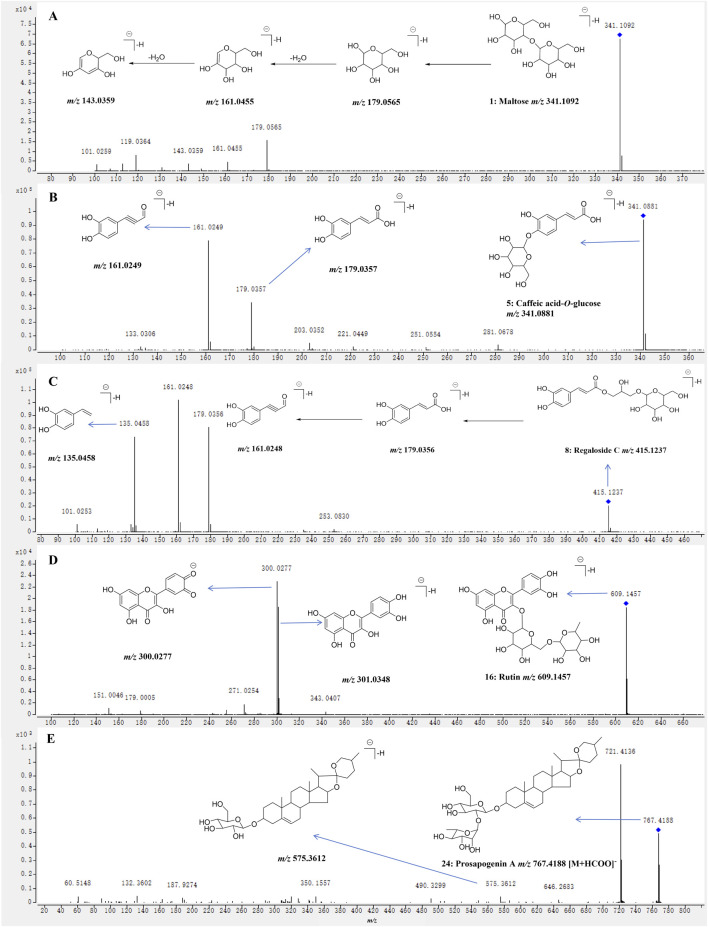
The MS/MS spectra and characteristic fragment ions of metabolites 1 **(A)**, 5 **(B)**, 8 **(C)**, 16 **(D)**, and 24 **(E)**.

The structural identification of metabolites **1**, **5**, **8**, **16**, and **24** is presented here to serve as an illustrative example. Metabolite **1** had a mass-to-charge ratio of *m/z* 341.1092 ([M-H]^-^). This *m/z* value was not reported in the lily metabolite database (Supplementary Table S1). The characteristic ions at *m/z* 341.1092, 179.0564, 161.0455, and 143.0359 were used for screening the potential metabolite in Massbank (https://massbank.eu/MassBank/). The result indicated that metabolite **1** was maltose. In its secondary mass spectrum, it lost a glucose molecule to form a fragment ion at *m/z* 179.0564. This fragment ion subsequently lost neutral H_2_O molecules to form ions at *m/z* 161.0455 and 143.0359. These fragment ions indicate that the ion at *m/z* 179.0564 is a deprotonated glucose. Therefore, metabolite **1** is a molecule containing two glucose units and was further identified as maltose (Figure 3A). This metabolite was identified in lilies for the first time. Metabolite **5** had a mass-to-charge ratio of *m/z* 341.0881 ([M-H]^-^), which was also not reported in the lily database (Supplementary Table S1). In its secondary mass spectrum, it lost a glucose molecule to form a fragment ion at *m/z* 179.0357. This fragment ion then lost a neutral H_2_O molecule to form *m/z* 161.0249. These fragments indicate that *m/z* 179.0357 is a deprotonated caffeic acid. Therefore, metabolite **5**, consisting of caffeic acid linked to a glucose molecule, was preliminarily identified as caffeic acid-*O*-glucoside (Figure 3B). This metabolite was identified in lilies for the first time. Metabolite **8** had a mass-to-charge ratio of *m/z* 415.1237 ([M-H]^-^). Database searching based on its *m/z* suggested it might be regaloside C. The MS/MS of metabolite **8** showed that the parent ion (*m/z* 415.1237) lost a glucose molecule to form a fragment ion at *m/z* 179.0356. This fragment ion then underwent neutral loss of H_2_O to form *m/z* 161.0248. These characteristic fragment ions are fully consistent with the mass spectrometric fragmentation behavior of regaloside C (Wang, et al., 2023), therefore, metabolite **8** was identified as regaloside C (Figure 3C). Metabolite **16** had a mass-to-charge ratio of *m/z* 609.1457 ([M-H]^-^). Database searching revealed this *m/z* was not reported in the lily database. Its characteristic fragment ions at *m/z* 301.0348, 300.0277, and 151.0046 indicated that metabolite **16** is rutin (Figure 3D), which was consistent with the result of Massbank. This is a common flavonoid in plants and was reported in lilies for the first time. Metabolite **24** had a mass-to-charge ratio of *m/z* 767.4188 ([M + HCOO]^-^). Database searching based on its *m/z* suggested it might be prosapogenin. In its secondary mass spectrum, it lost a molecule of HCOOH to form *m/z* 721.4236. This ion then lost a glucose molecule to form a fragment ion at *m/z* 575.1632. These characteristic fragment ions and the fragmentation pattern highly match those of prosapogenin. Therefore, metabolite **24** was preliminarily identified as prosapogenin (Figure 3E). Using structural identification methods similar to those described for the above five metabolites, the other 20 metabolites were identified in lilies (Table 1; Supplementary Figure S1).

**TABLE 1:** Metabolites identified in lily by UPLC-Q-TOF-MS technology.

No	*t*_R_ (min.)	[M- H]^-^ (*m/z*)	Molecular formula	MS/MS (*m/z*)	Distribution and relative abundance (%)	Tentative identification
1^a^	1.25	341.1092	C_12_H_22_O_11_	179.0565, 161.0455, 143.0359 119.0364, 101.0259	All^d^ (17.1, 21.3, 15.2, 16.4)	Maltose
2^a^	1.45	191.0203	C_6_H_8_O_7_	173.0098, 129.0198, 111.0094	All (12.0, 13.4, 11.8, 12.9)^e^	Citric acid
3^a^	1.85	191.0205	C_6_H_8_O_7_	173.0090, 129.0198, 111.0093	All (4.1, 3.5, 5.2, 5.6)	Isocitric acid
4^b^	4.83	179.0321	C_9_H_8_O_4_	135.0450, 117.0325, 107.0500	All (4.3, 1.2, 4.8, 4.6)	Caffeic acid
5^a^	6.52	341.0881	C_15_H_18_O_9_	179.0357, 161.0249	JD (0.2), LY (0.3), GYH (0.3)	Caffeic acid-*O*-glucose
6	7.21	325.0925	C_15_H_18_O_8_	163.0405, 145.0299	All (0.1, 0.1, 0.1, 0.1)	p-Hydroxy-Cinnamic acid-*O*-glucose
7	7.30	353.0881	C_16_H_18_O_9_	191.0567, 179.0363, 161.0264	All (0.2, 0.1, 0.2, 0.1)	Chlorogenic acid
8	7.50	415.1237	C_18_H_24_O_11_	179.0356, 161.0248, 135.0458	All (1.2, 1.1, 0.9, 1.3)	Regaloside C
9	7.60	741.1858	C_32_H_38_O_20_	579.1354, 285.0379	All (1.0, 0.8, 0.7, 1.2)	Demethyl-rutin-*O*-glucose
10	8.02	355.1039	C_16_H_20_O_9_	193.0510, 175.0408	All (1.5, 0.6, 1.2, 1.3)	Ferulic acid-*O*-glucose
11	8.10	399.1299	C_18_H_24_O_10_	163.0407, 145.0298	All (1.3, 0.4, 0.9, 1.0)	Regaloside H
12	8.31	253.0728	C_12_H_14_O_6_	179.0350, 161.0251, 135.0459	All (0.1, 0.1, 0.1, 0.1)	Caffeoylglycerol
13^b^	8.98	399.1292	C_18_H_24_O_10_	163.0405, 145.0301, 119.0497	All (6.6, 6.8, 6.2, 6.4)	Regaloside A
14	9.62	625.1408	C_27_H_30_O_17_	300.0274, 178.9998, 151.0052	All (0.1, 0.1, 0.1, 0.2)	Quercetin-*O*-diglucoside
15	10.31	237.0775	C_12_H_14_O_5_	163.0411, 145.0203, 119.0512	All (1.4, 1.6, 1.2, 1.3)	*p*-coumaroylglycerol
16^a^	10.95	609.1457	C_27_H_30_O_16_	300.0277, 271.0254, 151.0046	All (0.9, 0.1, 0.6, 0.5)	Rutin
17	12.01	579.1338	C_26_H_28_O_15_	285.0390, 284.0324, 151.0029	All (0.7, 0.1, 0.4, 0.6)	Demethyl-rutin
18^b^	12.42	441.1416	C_20_H_26_O_11_	399.1330, 381.1193, 163.0405 145.0299, 119.0499	All (8.3, 0.9, 2.7, 2.4)	Regaloside B
19^a^	13.57	623.1627	C_28_H_32_O_16_	315.0509, 299.0188, 151.0031	All (1.6, 2.7, 1.0, 1.2)	Methyl-rutin
20	13.59	693.2017	C_32_H_38_O_17_	517.1545, 337.0875, 175.0409	All (3.5, 3.1, 3.0, 3.2)	Diferuloylsucrose
21	15.97	723.2126	C_33_H_40_O_18_	517.1494, 205.0547	All (3.5, 2.9, 2.8, 2.7)	Methoxyl-diferuloylsucrose
22^c^	18.10	901.4684	C_45_H_70_O_16_	755.4136, 179.0621, 163.0603	JD (4.0), GYH (1.1)	Dehydroxy-dioscin
23^c^	18.45	903.4843	C_45_H_72_O_16_	757.4264, 163.0636	JD (4.6), GYH (0.9)	Dioscin
24^c^	20.8	767.4188	C_39_H_62_O_12_	721.4136, 575.3612, 187.9274	LY (4.2), GYH (1.1)	Prosapogenin A
25^c^	28.9	783.4122	C_39_H_62_O_13_	737.4081, 205.0722, 163.0610	LY (7.1), GYH (2.6)	Diosgenin-*O*-diglucoside

^a^ The metabolite was reported for the first time in lily.

^b^ The metabolite was unambiguously identified by comparing with the standards.

^c^ [M + HCOO^**−**^]^**-**^.

^d^ Represent the metabolite was detected in four lily extract.

^e^ Represent the relative abundance (compared with the total peak area of the metabolites) of identified compound in JD, LZ, LY, and LYH, extract, respectively; JD: *juandan* lily extract; LY: *longya* lily extract; GYH: *guiyanghong* lily extract.

**FIGURE 4:**
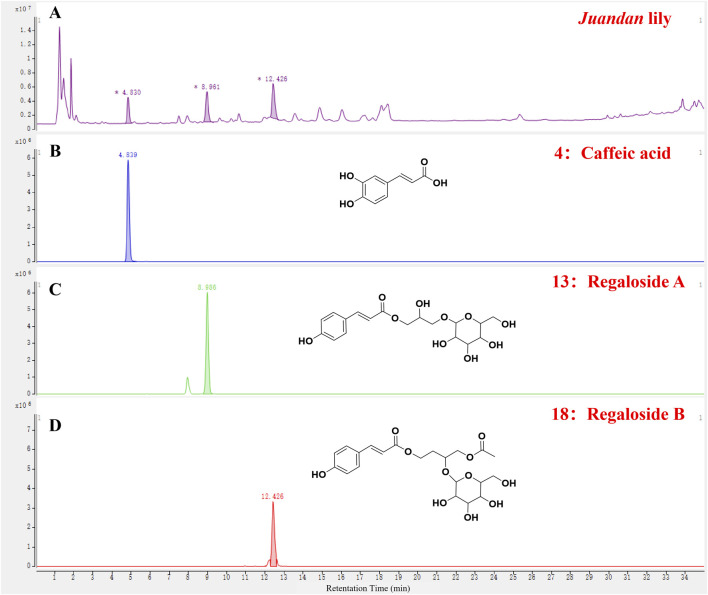
Identification of caffeic acid, regaloside A, and regaloside B in *Juandan* lily extract. Total ion chromatogram of *Juandan* lily extract **(A)**, caffeic acid **(B)**, regaloside A **(C)**, and regaloside B **(D)**.

Based on the aforementioned research, *Juandan* lily possesses significant hypoglycemic activity. What are its specific active hypoglycemic metabolites? Using mass spectrometry, three high-abundance metabolites (4, 13, and 18) were screened from the TIC of the Juandan lily extract, which may be the active hypoglycemic metabolites. To further confirm their structures, their retention times, primary and secondary mass spectra were compared with those of standards. Ultimately, metabolites 4, 13, and 18 were unambiguous identified as caffeic acid, regaloside A, and regaloside B, respectively (Figure 4, The UPLC-Q-TOF-MS method was the same as that used for the metabolite analysis of the extracts described previous). Subsequent studies were conducted on the hypoglycemic activities of caffeic acid, regaloside A, and regaloside B to determine whether they are the active hypoglycemic metabolites of *Juandan* lily.

Based on the above research, the primary metabolites in *Lilium* species are regaloside derivatives (e.g., metabolites 13 and 18) and dioscin-type steroidal saponins (e.g., metabolites 24 and 25). In addition, significant differences in chemical composition exist among different *Lilium* varieties. Both the number and content of metabolites in *Lanzhou* lily are lower than those in the other three varieties. Therefore, traditionally, *Lanzhou* lily is mainly used as food, whereas *Juandan* lily and *Longya* lily are primarily employed for medicinal purposes (Tang et al., 2026). Although a total of 20 identified metabolites are present in all four *Lilium* species, the contents of some metabolites vary considerably. For example, metabolite 18 (regaloside B) is predominantly found in Juandan lily, while its content is relatively low in the other three varieties. Metabolites 24 (prosapogenin A) and 25 (Diosgenin-*O*-diglucoside) are mainly present in *Longya* lily, but are rarely detected in *Juandan* lily and *Longya* lily. These steroidal saponins are closely associated with the bitterness of *Lilium* (Zhong et al., 2026) as well as its antioxidant, anti-inflammatory, antidepressant, and sleep-improving effects (Munafo et al., 2015).

### 3.3 The hypoglycemic activity of caffeic acid, regaloside A, and regaloside B

**FIGURE 5:**
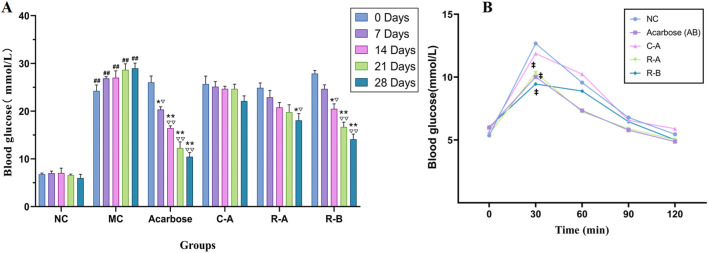
Effects of the three metabolites on blood glucose levels in hyperglycemic model mice **(A)**, n = 8–10) and postprandial blood glucose in mice (B, n = 10). ^##^ *p* < 0.01 compared the data with the normal control group in the same period; ^*^ *p* < 0.05, ^**^ *p* < 0.01 compared with the data at 0 days, ^▽^ *p* < 0.05, ^▽▽^ *p* < 0.01 compared the data with the model group in the same period; ^‡^ *p* < 0.05 compared with the data of NC at 30 min; NC: normal control group; MC: model control group; C–A: caffeic acid; R–A: regaloside A; R–B: regaloside **(B)**.

Caffeic acid, regaloside A, and regaloside B are the major constituents of *Juandan* lily extract. Are they the active hypoglycemic metabolites? As shown in Figure 5A, the blood glucose levels of the model group mice at day 0 (24.23 ± 1.26 mmol/L), day 7 (26.87 ± 0.35 mmol/L), day 14 (27.00 ± 1.45 mmol/L), day 21 (28.67 ± 1.24 mmol/L), and day 28 (28.97 ± 1.09 mmol/L) were significantly higher than those of the normal control group (*p* < 0.01), indicating the successful establishment of a hyperglycemic mouse model. The acarbose group exhibited significant hypoglycemic activity, with blood glucose levels decreasing from an initial values of 26.03 ± 1.34 mmol/L to 20.34 ± 0.57 (day 7, *p* < 0.05), 16.45 ± 0.45 (day 14, *p* < 0.05), 12.24 ± 1.32 mmol/L (day 21, *p* < 0.01), and 10.45 ± 0.87 mmol/L (day 28, *p* < 0.01). Regaloside B demonstrated significant hypoglycemic activity, reducing blood glucose levels from an initial values of 27.87 ± 0.67 mmol/L (day 0) to 20.49 ± 1.04 (day 14, *p* < 0.05), 16.67 ± 1.03 mmol/L (day 21, *p* < 0.01), and 14.11 ± 1.09 mmol/L (day 28, *p* < 0.01). Its hypoglycemic activity was comparable to that of acarbose. Compared to the model group, regaloside A also significantly lowered blood glucose levels in mice, decreasing from 24.89 ± 1.02 mmol/L (day 0) to 18.07 ± 1.43 mmol/L (day 28, *p* < 0.05). In contrast, caffeic acid did not exhibit hypoglycemic activity. Therefore, regaloside A and regaloside B are the active hypoglycemic metabolites in *Juandan* lily, with regaloside B showing stronger activity than regaloside A.

As shown in Figure 5B, acarbose exhibited significant activity in reducing postprandial blood glucose, demonstrating a notable decrease compared to the normal control group at 30 min postprandial (*p* < 0.05). Both regaloside A and regaloside B also showed activity in lowering postprandial blood glucose (*p* < 0.05), with regaloside B exhibiting stronger activity than regaloside A. The postprandial blood glucose-lowering activity of regaloside B was consistent with that of acarbose. Caffeic acid also demonstrated certain activity in reducing postprandial blood glucose, though its effect was weaker than that of regaloside A and B. The above experimental results indicate that regaloside A, and regaloside B display significant activity in lowering postprandial blood glucose in normal mice.

### 3.4 The α-glucosidase inhibitory activity of four lily extracts, caffeic acid, regaloside A, and regaloside B

**FIGURE 6:**
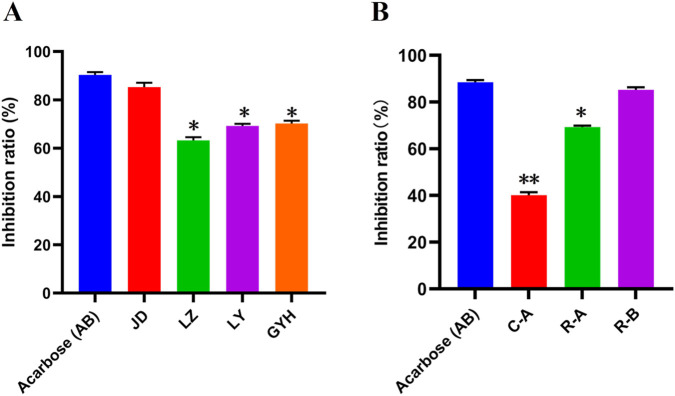
Investigation of α-Glucosidase inhibitory activity of four lily extracts, caffeic acid, regaloside **(A)**, and regaloside B (n = 3). JD, *Juandan* lily; LZ, *Lanzhou* lily; LY, *Longya* lily; GYH, *Guiyanghong* lily; C-A, caffeic acid; R-A, Regaloside A; R-B, Regaloside **(B)** ^*^ *p* < 0.05, ^**^ *p* < 0.01 compared with the data of acarbose.

The extracts of *Juandan* Lily, *Lanzhou* Lily, *Longya* Lily, and *Longyahong* Lily exhibit hypoglycemic activity. What is the underlying mechanism? The study on α-glucosidase inhibitory activity revealed that at a concentration of 4 mg/mL, the inhibition rates of acarbose and the extracts of four lilies were 90.34% ± 1.12%, 85.27% ± 1.81%, 63.23% ± 1.25%, 69.21% ± 0.89%, and 70.23% ± 1.12%, respectively. The extracts of four lilies displayed inhibitory effects on α-glucosidase, with the *Juandan* Lily extract showing the strongest inhibitory activity than the other three lily extracts (Figure 6A). The inhibition rates of acarbose was significant higher than that of *Lanzhou* Lily, *Longya* Lily, and *Longyahong* Lily extracts (*p* < 0.05). Furthermore, the IC_50_ values of acarbose and the *Juandan* Lily extract against α-glucosidase to be 2.94 and 3.11 mg/mL, respectively, indicating that the inhibitory activity of the *Juandan* Lily extract is comparable to that of the positive control drug.

Caffeic acid, regaloside A, and regaloside B exhibit activity in lowering postprandial blood glucose in mice. What is the potential mechanism? Investigation of α-glucosidase inhibitory activity revealed that at a concentration of 4 mg/mL, the inhibition rates of caffeic acid, regaloside A, and regaloside B were 40.14% ± 1.21%, 69.31% ± 0.57%, and 85.21% ± 1.04%, respectively, while the inhibition rate of acarbose was 88.45% ± 0.89%. This indicates that regaloside A and regaloside B significantly inhibit α-glucosidase, with regaloside B demonstrating a stronger inhibitory effect than regaloside A (Figure 6B). The inhibition rates of acarbose was significant higher than that of caffeic acid (*p* < 0.01), regaloside A (*p* < 0.01). Furthermore, the IC_50_ values of acarbose and regaloside B against α-glucosidase to be 2.78 and 2.99 mg/mL, respectively, suggesting that the inhibitory activity of regaloside B is consistent with that of acarbose.

In summary, the extracts of *Juandan* Lily, *Lanzhou* Lily, *Longya* Lily, and *Longyahong* Lily possess hypoglycemic activity. Their potential mechanism lies in inhibiting intestinal α-glucosidase activity, thereby attenuating the postprandial rise in blood glucose (IC_50_: 3.11 mg/mL for *Juandan* Lily extract). Regaloside B, a major active metabolite of *Juandan* Lily, also exhibits significant α-glucosidase inhibitory activity (IC_50_: 2.99 mg/mL), contributing to its potential hypoglycemic mechanism.

In previous studies, crude polysaccharides or total steroidal saponin extracts from *Lily* were found to possess hypoglycemic activity (Zhu et al., 2013; Zhang et al., 2025); however, the specific hypoglycemic active metabolites have remained unknown. In the present study, we first evaluated the hypoglycemic activity of extracts from four different *Lilium* varieties and found that the extract of *Juandan* lily exhibited the strongest hypoglycemic effect. Subsequently, mass spectrometry was employed to identify the major metabolites in *Juandan* lily, revealing three metabolites with relatively high content: caffeic acid, regaloside A, and regaloside B. The hypoglycemic activity of these three metabolites was then evaluated, and the results showed that regaloside A and regaloside B possess significant hypoglycemic activity, with regaloside B exhibiting the strongest effect. This study is the first to elucidate that the hypoglycemic active metabolites in *Juandan* lily are regaloside A and regaloside B. However, there are several other metabolites with relatively high content in the TIC of *Juandan* lily, such as metabolites **1**–**3**. Whether these are potential hypoglycemic active metabolites requires further investigation.

### 3.5 Molecular docking of acabose, regaloside A and B with α-glucosidase (maltase-glucoamylase and sucrase-isomaltase)

**FIGURE 7:**
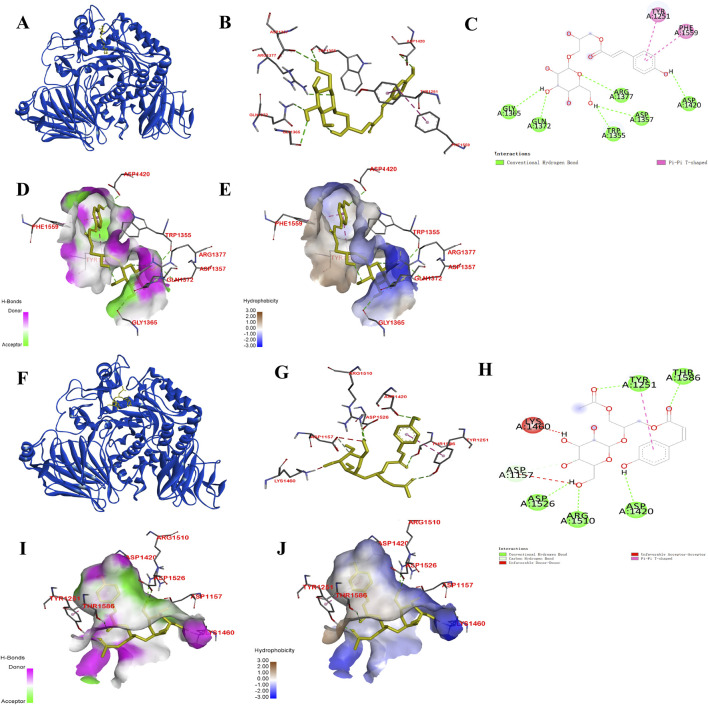
Molecular docking of regaloside **(A,B)** with human maltase-glucoamylase. Overall structure of regaloside **(A,B)** with maltase-glucoamylase **(A,F)**. The 3D **(B,G)** and 2D images **(C,H)** of regaloside **(A,B)** with the amino acid residues of maltase-glucoamylase. The 3D images **(D,I)** of hydrogen bond acceptor (Green) and donor (Purple). The 3D images **(E,J)** of hydrophobic interaction.

The α-glucosidase enzymes in the human intestine mainly include maltase-glucoamylase and sucrase-isomaltase, and these two enzymes primarily possess N- and C-terminal subunit active sites. To evaluate the interactions between regaloside A and B with α-glucosidase, molecular docking was employed to analyze their binding capabilities with the N- and C-terminal subunit of maltase-glucoamylase and sucrase-isomaltase. In this study, molecular docking was first performed between acarbose, regaloside A and regaloside B with the C-, N-terminal subunit of human maltase-glucoamylase. It was found that the binding capabilities of acarbose (−9.1 *vs* −7.3 kcal/mol, Supplementary Figures S2,S3), regaloside A (−7.7 *vs* −7.3 kcal/mol, Figure 7A; Supplementary Figure S4), and regaloside B (−7.9 *vs* −7.2 kcal/mol, Figure 7F; Supplementary Figure S4) to the C-terminal subunit was stronger than that to the N-terminal subunit. These docking results indicate that both the N-terminal and C-terminal subunits are active sites of human maltase-glucoamylase. Furthermore, molecular docking was performed between acarbose, regaloside A and regaloside B with the N-terminal subunit of human sucrase-isomaltase (docking with the C-terminal subunit could not be performed due to lack of data). The results showed that acarbose (−6.9 kcal/mol, Supplementary Figure S5), regaloside A (−6.7 kcal/mol, Supplementary Figure S6), and regaloside B (−6.9 kcal/mol, Supplementary Figure S6) formed complexes with the N-terminal subunit of human sucrase-isomaltase. In conclusion, acarbose, regaloside A, and regaloside B can interactive with the C- and N-terminal subunits of human maltase-glucoamylase and the N-terminal subunit of human sucrase-isomaltase, thereby may inhibiting postprandial blood glucose elevation.

In this study, the docking of regaloside A and regaloside B with the C-terminal subunit of human maltase-glucoamylase is used as a specific example to illustrate the binding situation between the compounds and the target protein. From an overall conformational perspective, regaloside A (Figure 7B) and B (Figure 7G) are embedded within the cavity of human maltase-glucoamylase, suggesting that they may exert inhibitory effects through competitive binding. Three-dimensional structural analysis revealed that regaloside A and B interact with multiple key amino acid residues within the active pocket of maltase-glucoamylase. In terms of hydrogen bonding, regaloside A forms multiple hydrogen bonds with residues such as Trp1355, Asp1357, Gly1365, Gln1372, Arg1377, and Asp1420 in maltase-glucoamylase (Figures 7C,D), indicating that this metabolite can directly target the catalytic core region of α-glucosidase enzyme, thereby potentially interfering with the substrate hydrolysis process. Meanwhile, regaloside B forms multiple hydrogen bonds with residues including Tyr1251, Asp1420, Arg1510, Asp1526, and Thr1586 in maltase-glucoamylase (Figures 7H,I), thereby acting on the catalytic core region and disrupting the catalytic function. Hydrophobic interaction analysis showed that the aromatic benzene ring structures in regaloside A and B form π-π T-shaped interactions with Tyr1251, further enhancing the binding capability of regaloside A and B to maltase-glucoamylase (Figures 7E,J). In summary, regaloside A and B adopt a hydrogen bond-dominated interaction mode within the active pocket, supplemented by π-π interactions, forming complexes with maltase-glucoamylase, thereby may inhibiting its activity and contributing to postprandial blood glucose reduction. This study provides a theoretical basis at the molecular docking level for the potential hypoglycemic mechanisms of the *Juandan* lily extract, regaloside A, and regaloside B.

Traditional Chinese medicine or natural products do not treat T2DM through a single target but operate via an integrated regulatory model of “multi-component, multi-target, multi-pathway”, exerting comprehensive therapeutic effects on its complex pathophysiology (Zhang et al., 2024; Ma et al., 2025; Zhang et al., 2025; Zhuang et al., 2025). In the present study, we only investigated the inhibitory activity of *Lilium* extracts, regaloside A, and regaloside B against α-glucosidase, and preliminarily elucidated their potential mechanisms of action through molecular docking techniques. However, there are two important targets in glycemic control—monophosphate-activated protein kinase (AMPK) and dipeptidyl peptidase-4 (DPP-4) (Malmgren, et al., 2015; Shrestha et al., 2016; Herrera-Balandrano et al., 2021). By activating AMPK activity or inhibiting DPP-4 enzyme activity, the extract or metabolite promotes glucose uptake and utilization, thereby reducing blood glucose levels in the body (Chang et al., 2025; Gong, et al., 2024; Xiong et al., 2025). Whether the hypoglycemic activity of lily extracts, regaloside A, and regaloside B is achieved through their effects on the AMPK and DPP-4 requires further in-depth investigation.

## 4 Conclusion

This study provides the first comparative evaluation of the hypoglycemic activity of *Juandan* lily, *Lanzhou* lLily, *Longya* lily, and *Longyahong* lily. The results demonstrate that the extract of *Juandan* lily possesses the most potent hypoglycemic activity, significantly reducing blood glucose levels in hyperglycemic model mice and lowering postprandial blood glucose in normal mice. The extract exerts its effect by inhibiting α-glucosidase activity, thereby attenuating the postprandial rise in blood glucose. Based on UPLC-Q-TOF-MS analysis, 25 metabolites with relatively high content were identified from the lily extracts, among which six were identified for the first time. Caffeic acid, regaloside A, and regaloside B were identified as the major constituents of *Juandan* lily. Subsequent investigation of their hypoglycemic activity revealed that regaloside A and B exhibit significant effects, markedly lowering blood glucose levels in hyperglycemic model mice and reducing postprandial blood glucose in normal mice. *In vitro* α-glucosidase inhibition assays and molecular docking studies indicate that the hypoglycemic mechanisms of regaloside A and B involve inhibiting intestinal α-glucosidase. Notably, regaloside B demonstrates stronger hypoglycemic activity and *in vitro* α-glucosidase inhibitory potency compared to positive control acarbose. This research lays a foundation for the development of novel hypoglycemic drugs or functional foods and promotes the high-value utilization of lily resources.

## Data Availability

The original contributions presented in the study are included in the article/Supplementary Material, further inquiries can be directed to the corresponding authors.
