# Supplementary material for: The hypoglycemic metabolites and potential mechanisms of Lilium lancifolium (Juandan lily)
Source: Front Pharmacol. 2026 Jun 8;17:1806579. doi: 10.3389/fphar.2026.1806579 (PMC13283800; doi:10.3389/fphar.2026.1806579)

Supplementary Material

# Table S1 The reported compounds in previously studies from Lily.

| No. | Compound | Molecular Formula | Structure | Molecular weight | [M+H]^+^ | [M-H]^-^ |
| --- | --- | --- | --- | --- | --- | --- |
|  | Deacyl-brownioside | C_39_H_62_0_15_ |  | 770.4089 | 771.4161 | 771.4172 |
|  | 27-O-（3- hydroxy- 3- methylglutaroyl）spirost- 5- ene- 3β，27- diol-（isonarthogenin）- 3- O- α- L- rha-（1→2）- O- [ β- D- glu-（1→4）]- β- D- glucoside | C_50_H_78_O_25_ |  | 1078.4823 | 1079.4905 | 1079.4916 |
|  | 27- O- [（3S）- 3- O- β- D- glu- 3- methylglutaroyl]isonarthogenin- 3- O- [α- L- rha-（1→2）]- β- D- glucoside | C_50_H_78_O_23_ |  | 1046.4934 | 1047.5007 | 1047.5018 |
|  | （24S，25S）- 3β，17α，24- trihydroxy- 5α- spirostan- 6- one- 3- O- [α- L- rha-（1→2）]- β- D- glucoside | C_39_H_62_O_17_ |  | 802.3987 | 803.4060 | 803.4071 |
|  | （25R）- 3β- hydroxy- 5α- spirostan- 6- one- 3- O- α- L- rha-（1→2）- β- D- glucoside | C39H62O15 |  | 770.4089 | 771.4161 | 771.4172 |
|  | （25R）- 3β，17α- dihydroxy-5α- spirostan-6-one- 3-O-α- L-rha-（1→2）-β-D- glucoside | C_39_H_62_O_16_ |  | 786.4038 | 787.4111 | 787.4122 |
|  | Pumilum A | C_39_H_62_O_17_ |  | 802.3987 | 803.4060 | 803.4071 |
|  | （25R）- 3β，17α- dihydroxy- 5α- spirostan- 6- one- 3- O- α- L- rha-（l→2）- O- [α- L- ara-（l→3）]- β- D- glucoside | C_46_H_74_O_22_ |  | 978.4672 | 979.4745 | 979.4755 |
|  | （25R）- 3β- hydroxy- 5α- spirostan- 6- one- 3- O- α- L- ara-（1→6）- β- D- glucoside | C_40_H_64_O_16_ |  | 800.4194 | 801.4267 | 801.4278 |
|  | （25R）- 27- O- [（S）- 3- hydroxy- 3- methylglutaryl]- spirost- 5α- 3β，27- diol- 3- O- α- L- rha-（1→2）- β- D- glucoside | C_43_H_68_O_19_ |  | 888.4355 | 889.4428 | 889.4439 |
|  | （25R）- 5α- spirostan- 3β，17α- diol- 3- O- β- D- xyl-（1→4）- [α- L- ara-（1→6）]- β- D- glucoside | C_45_H_74_O_20_ |  | 934.4773 | 935.4846 | 935.4857 |
|  | Brownioside | C_43_H_66_O_20_ |  | 902.4147 | 903.4220 | 903.4231 |
|  | （25R）- spirost- 5- ene- 3β- O- α- L- rha-（1→2）- [β- D- glu-（1→6）]- β- D- glucoside | C_45_H_72_O_20_ |  | 932.4617 | 933.4690 | 933.4701 |
|  | （25R，26R）- 26- methoxyspirost- 5- ene- 3β- O- α- L- rha-（1→2）- [β- D- glu-（1→6）]- β- D- glucoside | C_46_H_74_O_21_ |  | 962.4723 | 963.4795 | 963.4806 |
|  | （25R，26R）- 17α- hydroxy- 26- methoxyspirost- 5- ene- 3β- O- α- L- rha-（1→2）- [β- D- glu-（1→6）]- β- D- glucoside | C_47_H_78_O_22_ |  | 994.4985 | 995.5058 | 995.5068 |
|  | Dioscin | C_45_H_74_O_19_ |  | 918.4824 | 919.4897 | 919.4908 |
|  | Ophiopogonin D | C_44_H_74_O_19_ |  | 902.4511 | 903.4584 | 903.4595 |
|  | Lililancifoloside A | C_45_H_72_O_19_ |  | 916.4668 | 917.4741 | 917.4752 |
|  | 26-O-β-D-glu- nuatigenin | C_33_H_54_O_10_ |  | 610.3717 | 611.3790 | 611.3801 |
|  | 26-O-β-D-glu- nuatigenin-3-O-β-D- glucoside | C_39_H_64_O_16_ |  | 788.4194 | 789.4267 | 789.4278 |
|  | 26-O-β-D-glu- nuatigenin-3-O-[α- L-rha-（1→2）]-β- D-glucoside | C_45_H_74_O_21_ |  | 950.4723 | 951.4795 | 951.4806 |
|  | 26- O- β- D- glu- nuatigenin- 3- O- α- L- rha-（1→2）- O- [β- D- glu-（1→4）]- β- D- glucoside | C_51_H_84_O_27_ |  | 1128.5200 | 1129.5273 | 1129.5284 |
|  | 26- O- β- D- glu- nuatigenin- 3- O- α- L- rha-（1→2）- [β- D- glu-（1→6）]- β- D- glucoside | C_51_H_84_O_27_ |  | 1128.5200 | 1129.5273 | 1129.5284 |
|  | 26- O- [β- D- glu-（1→2）]- β- D- glu- nuatigenin- 3- O- [α- L- rha-（1→2）]- β- D- glucoside | C_51_H_84_O_27_ |  | 1128.5200 | 1129.5273 | 1129.5284 |
|  | （25R）- 26- O- β- D- glu- 22α- hydroxyfurost- 5- en- 3β- O- α- L- ara-（1→3）- O- [β- D- glu-（1→4）]- O- [α- L- rha-（1→2）] - β- D- glucoside | C57H94O32 |  | 1290.5728 | 1291.5801 | 1291.5812 |
|  | （25R）-26- O-β-D- glu- 22α- hydroxyfurost- 5- en- 3β- O- α- L- ara-（1→4）- O- [β- D- glu-（1→3）]- O- [α- L- rha-（1→2）] - β- D- glucoside | C_57_H_94_O_32_ |  | 1290.5728 | 1291.5801 | 1291.5812 |
|  | （25R）- 26- O- β- D- glu- furosta- 5，20（22）- dien- 3β- O- α- L- ara-（1→3）- O- [β- D- glu-（1→4）]- O- [α- L- rha-（1→2）] - β- D- glucoside | C_58_H_94_O_32_ |  | 1302.5728 | 1303.5801 | 1303.5812 |
|  | （25R）- 26- O- β- D- glu- furosta- 5，20（22）- dien- 3β- O- α- L- ara-（1→4）- O- [β- D- glu-（1→3）]- O- [α- L- rha-（1→2）] - β- D- glucoside | C_58_H_94_O_32_ |  | 1302.5728 | 1303.5801 | 1303.5812 |
|  | （25R）- 26- O- β- D- glu- furost- 5- en- 3β，22α，26- triol- 3- O- α- L- rha-（1→2）- [β- D- glu-（1→4）]- β- D- glucoside | C_51_H_84_O_27_ |  | 1128.5200 | 1129.5273 | 1129.5284 |
|  | Tenuifolioside A | C_33_H_56_O_11_ |  | ^628.3823^ | 629.3895 | 629.3906 |
|  | Tenuifolioside B | C_30_H_50_O_5_ |  | 490.3658 | 491.3731 | 491.3742 |
|  | tenuifoliol 3-O-[β- D-glu-（1→4）]-β-D-glucoside | C_39_H_66_O_17_ |  | 806.4300 | 807.4373 | 807.4384 |
|  | 26-O-β- D-glu-3β，26-dihydroxycholestan-16,22-dioxo-3-O- α-rha（1→2-β-D- glucoside | C_45_H_74_O_21_ |  | 950.4723 | 951.4715 | 951.4806 |
|  | 26- O- β- D- glu- 3β，26- dihydroxy- 5- cholesten- 16，22- dioxo- 3- O- α- rha（1→2）- β- D- glucoside | C_45_H_72_O_21_ |  | 948.4566 | 949.4639 | 949.4650 |
|  | β-sitosterol | C_29_H_50_O |  | 414.3862 | 415.3934 | 415.3945 |
|  | Daucosterol | C_35_H_60_O_7_ |  | 592.4339 | 593.4412 | 593.4423 |
|  | Stigmasterol | C_29_H_48_O |  | 412.3705 | 413.3778 | 413.3789 |
|  | Stigmasterol- 3- β- D-glucoside） | C_35_H_58_O_7_ |  | 590.4183 | 591.4255 | 591.4266 |
|  | 1- O- caffeoylglycerol | C_12_H_14_O_6_ |  | ^254.0790^ | 255.0863 | 255.0874 |
|  | 1- O- feruloylglycerol | C_13_H_16_O_6_ |  | 268.0947 | 269.1020 | 269.1031 |
|  | 1- O- p- coumaroylglycerol | C_12_H_14_O_5_ |  | 238.0841 | 239.0914 | 239.0925 |
|  | 1,2- O- diferuloylglycerol | C_23_H_24_O_9_ |  | 444.1420 | 445.1493 | 445.1504 |
|  | 1,3- O- diferuloylglycerol | C_22_H_22_O_9_ |  | 430.1264 | 431.1337 | 431.1348 |
|  | 1,3- O- di-p-coumaroylglycerol | C_20_H_18_O_7_ |  | 370.1053 | 371.1125 | 371.1136 |
|  | 1- O-caffeoyl- 3- O- p-coumaroylglycerol | C_20_H_18_O_8_ |  | 386.1002 | 387.1074 | 387.1085 |
|  | 1- O- feruloyl- 3- O- p- coumaroylglycerol | C_21_H_20_O_8_ |  | 400.1158 | 401.1231 | 401.1242 |
|  | 1- O- feruloyl- 2- O- p- coumaroylglycerol | C_22_H_22_O_8_ |  | 414.1315 | 415.1387 | 415.1398 |
|  | 1- O- p-coumaroyl-2-O-feruloylglycerol | C_22_H_22_O_8_ |  | 414.1315 | 415.1387 | 415.1398 |
|  | Regaloside A | C_19_H_26_O_10_ |  | 414.1526 | 415.1599 | 415.1610 |
|  | Regaloside B | C_19_H_24_O_11_ |  | 428.1319 | 429.1391 | 429.1402 |
|  | Regaloside C | C_18_H_24_O_11_ |  | 416.1319 | 417.1391 | 417.1402 |
|  | Regaloside D | C_18_H_24_O_10_ |  | 400.1369 | 401.1442 | 401.1453 |
|  | Regaloside E | C_20_H_26_O_12_ |  | 458.1424 | 459.1497 | 459.1508 |
|  | Regaloside F | C_19_H_26_O_11_ |  | 430.1475 | 431.1548 | 431.1559 |
|  | Rutin | C_27_H_30_O_18_ |  | ^642.1432^ | 643.1505 | 643.1516 |
|  | Quercetin | C_15_H_10_O_7_ |  | 302.0427 | 303.0499 | 303.0510 |
|  | Dihydroquercetin | C_15_H_10_O_7_ |  | 302.0427 | 303.0499 | 303.0510 |
|  | Kaempferol | C_15_H_10_O_6_ |  | 286.0477 | 287.0550 | 287.0561 |
|  | Myricetin | C_15_H_10_O_8_ |  | 318.0376 | 319.0448 | 319.0459 |
|  | Dihydromyricetin | C_15_H_10_O_8_ |  | 318.0376 | 319.0448 | 319.0459 |
|  | Eriodictyol | C_16_H_12_O_6_ |  | 300.0634 | 301.0707 | 301.0718 |
|  | Catechin | C_15_H_14_O_6_ |  | 290.0790 | 291.0863 | 291.0874 |
|  | Epicatechin | C_15_H_14_O_6_ |  | 290.0790 | 291.0863 | 291.0874 |
|  | Cyanidin 3-rutinoside | C_27_H_32_O_18_ |  | 644.1583 | 645.1656 | 645.1667 |
|  | Phloridzin | C_21_H_24_O_12_ |  | 468.1268 | 469.1341 | 469.1351 |
|  | *p*-Coumaric acid | C_9_H_8_O_3_ |  | 164.0473 | 165.0546 | 165.0557 |
|  | Ferulic acid | C_10_H_10_O_4_ |  | 194.0579 | 195.0652 | 195.0663 |
|  | Caffeic acid | C_9_H_8_O_4_ |  | 180.0423 | 181.0495 | 181.0506 |
|  | Chlorogenic acid | C_16_H_18_O_9_ |  | 354.0951 | 355.1024 | 355.1035 |
|  | Eugenol 4- O- α- L- rha-（1→6）- β- D- glucoside | C_23_H_34_O_13_ |  | 518.1999 | 519.2072 | 519.2083 |
|  | 2,6- dimethoxy- 4-（prop- 2- enyl）phenyl- O- α- L- rha-（1→6）- β- D- glucoside | C_23_H_34_O_14_ |  | 534.1949 | 535.2021 | 535.2032 |
|  | 2,6- dimethoxy- 4-（prop- 2- enyl）phenyl-O-β-D-glu-(1→6)-β-D- glucoside | C_23_H_34_O_15_ |  | 550.1898 | 551.1970 | 551.1981 |
|  | 3,6'-O- diferuloylsucrose | C_33_H_40_O_16_ |  | 692.2316 | 693.2389 | 693.2400 |
|  | Colchicine | C_22_H_25_NO_6_ |  | ^399.1682^ | 400.1755 | 400.1766 |
|  | β-lumicolchicine | C_23_H_27_O_6_ |  | 413.1838 | 414.1911 | 414.1922 |
|  | Berberine | C_20_H_18_NO_4_^+^ |  | 336.1230 | 337.1303 | 337.1314 |
|  | β1-solamargine | C_39_H_63_NO_13_ |  | 753.4299 | 754.4372 | 754.4383 |
|  | solasodine- 3- O- α- L- rha-（1→2）-O- [β- D- glu-（1→4）]- β-D-glucoside | C_45_H_73_NO_19_ |  | 931.4777 | 932.4850 | 932.4861 |
|  | （22R，25R）- spirosol- 5α- 3β- O- [α- L- rha-（1→2）]- β-D-glucoside | C_39_H_63_NO_13_ |  | 753.4299 | 754.4372 | 754.4383 |
|  | Adenosine | C_10_H_13_N_5_O_4_ |  | 267.0968 | 268.1040 | 286.1051 |
|  | 2'-deoxyadenosine | C_10_H_13_N_5_O_3_ |  | 251.1018 | 252.1091 | 252.1102 |
|  | n-butyl-β-D- fructopyranoside | C_10_H_20_O_6_ |  | 236.1260 | 237.1333 | 237.1344 |
|  | Methyl-β-D-fructofuranoside- | C_7_H_14_O_6_ |  | 194.0790 | 195.0863 | 195.0874 |
|  | Methyl-α-D-glucopyranoside | C_7_H_14_O_6_ |  | 194.0790 | 195.0863 | 195.0874 |
|  | Methyl- α-D-mannopyranoside） | C_7_H_14_O_6_ |  | 194.0790 | 195.0863 | 195.0874 |
|  | 2,4,6- trichlorol- 3- methyl- 5-methoxy- phenol- 1- O- β- D- glu-（1→6）- β- D-glucopyranoside | C_20_H_27_Cl_3_O_14_ |  | 596.0466 | 597.0539 | 597.0550 |
|  | 4- chlorol- 5-hydroxyl- 3-methyl- phenol- 1- O- α- L- rha（1→6）-β-D-glucopyranoside | C_20_H_29_ClO_13_ |  | 512.1297 | 513.1369 | 513.1380 |
|  | threo- 1-（4′-hydroxy-2′-methoxyphenyl）- 2-（2′′，4′′- dihydroxyphenyl）-1，3-propanediol- 4′- O- β-D-glucopyranoside | C_23_H_30_O_12_ |  | 498.1737 | 499.1810 | 499.1821 |
|  | Protocatechualdehyde | C_7_H_6_O_3_ |  | 138.0317 | 139.0390 | 139.0401 |
|  | *p*-hydroxybenzaldehyde | C_7_H_6_O_2_ |  | 122.0368 | 123.0441 | 123.0452 |
|  | Vanillic acid | C_8_H_8_O_3_ |  | 152.0473 | 153.0546 | 153.0557 |
|  | Salicylic acid | C_7_H_6_O_2_ |  | 122.0368 | 123.0441 | 123.0452 |
|  | Gallic acid | C_7_H_6_O_5_ |  | 170.0215 | 171.0288 | 171.0299 |
|  | Syringic acid | C_9_H_10_O_5_ |  | 198.0528 | 199.0601 | 199.0612 |
|  | Eicosanoic acid | C_20_H_40_O_2_ |  | 312.3028 | 313.3101 | 313.3112 |
|  | Heneicosanoic acid | C_21_H_42_O_2_ |  | 326.3185 | 327.3258 | 327.3269 |
|  | N-docosane acid | C_22_H_44_O_2_ |  | 340.3341 | 341.3414 | 341.3425 |
|  | Nonacosanol | C_29_H_60_O |  | 424.4644 | 425.4717 | 425.4728 |
|  | n-tetratriacontanol | C_34_H_70_O |  | 494.5427 | 495.5499 | 495.5510 |

Fig. S1 the MS/MS spectra of compounds **1**-**25**.


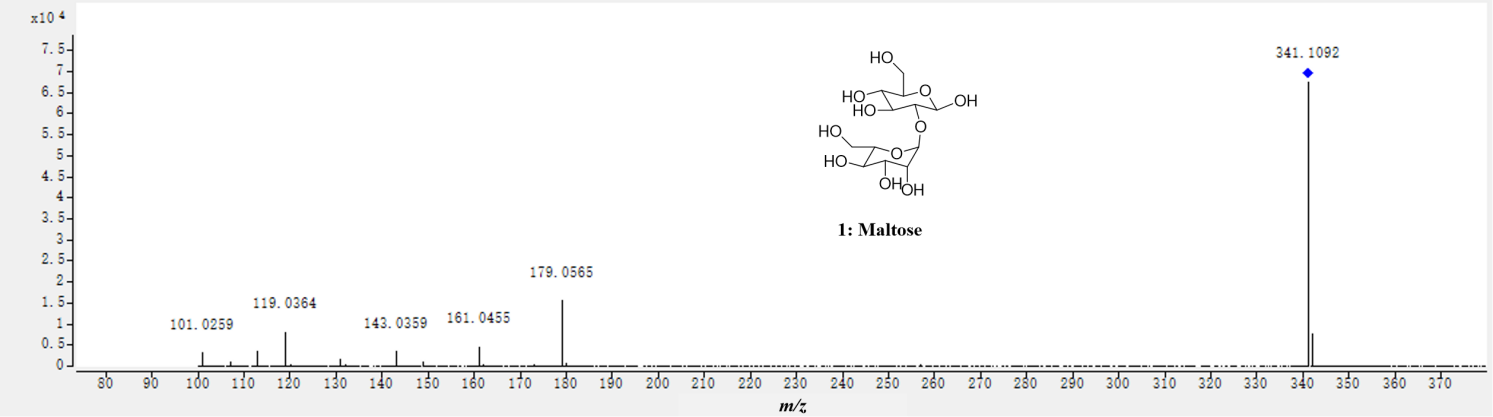


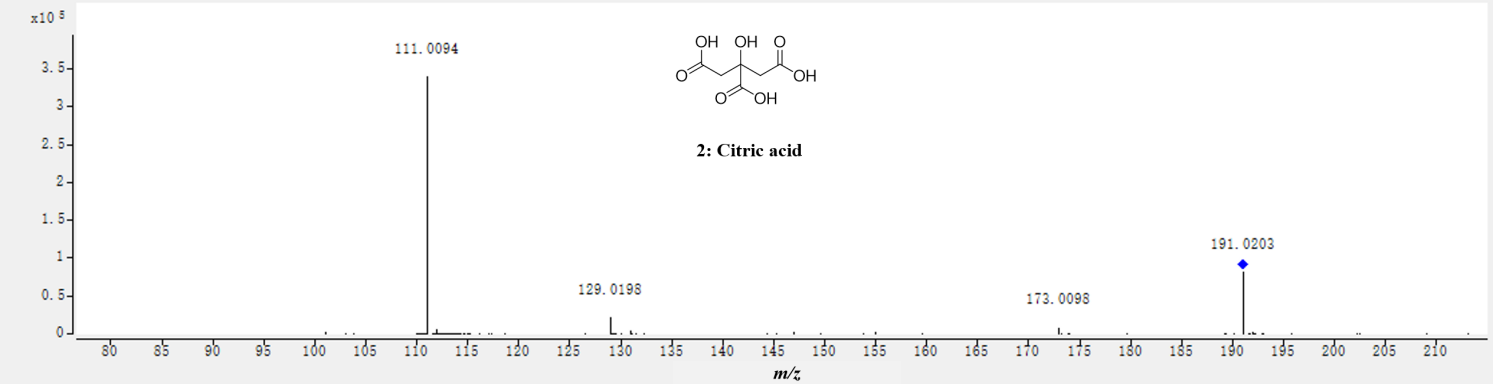


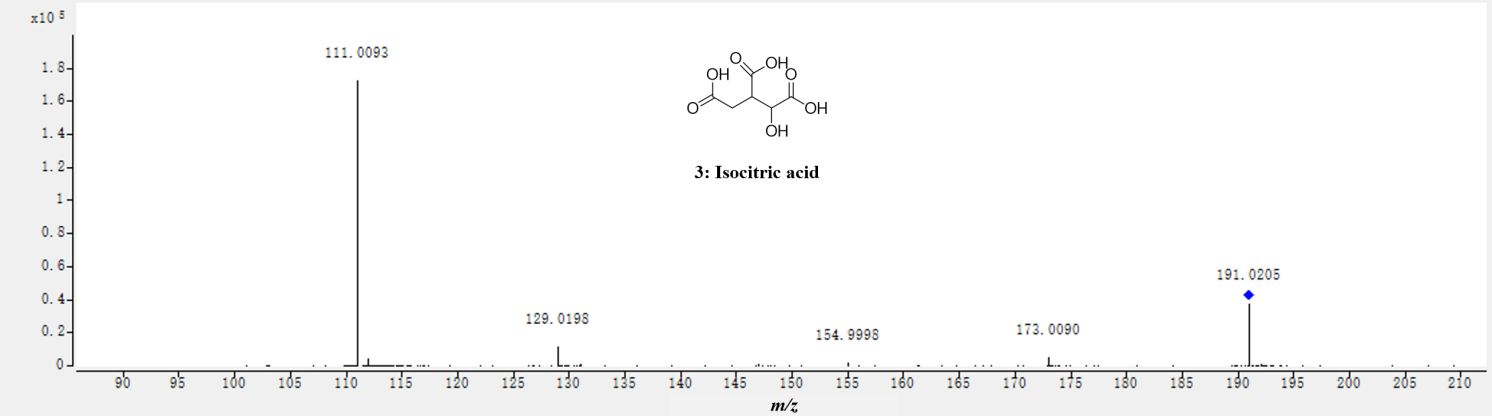


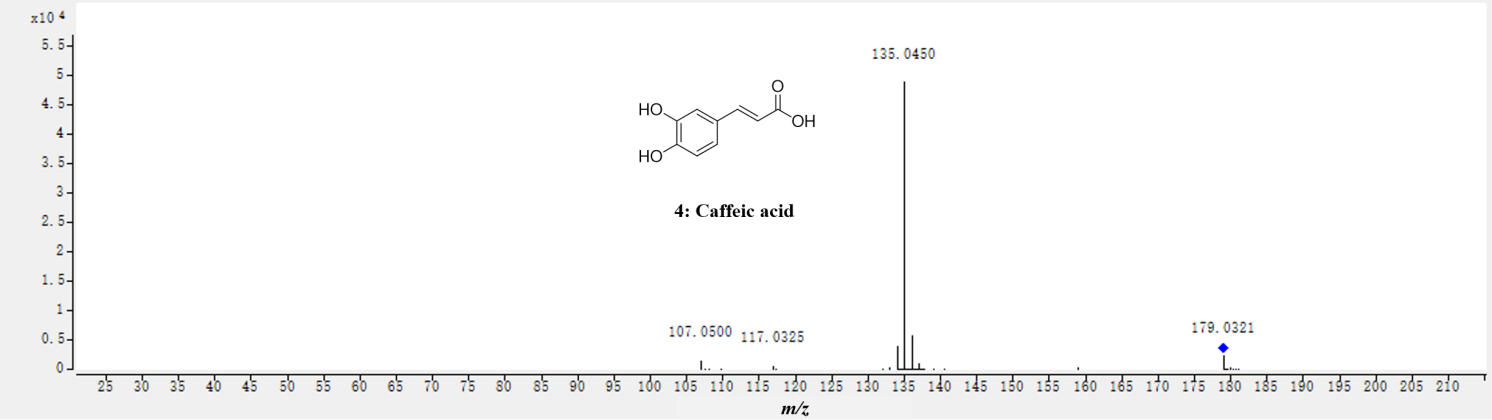


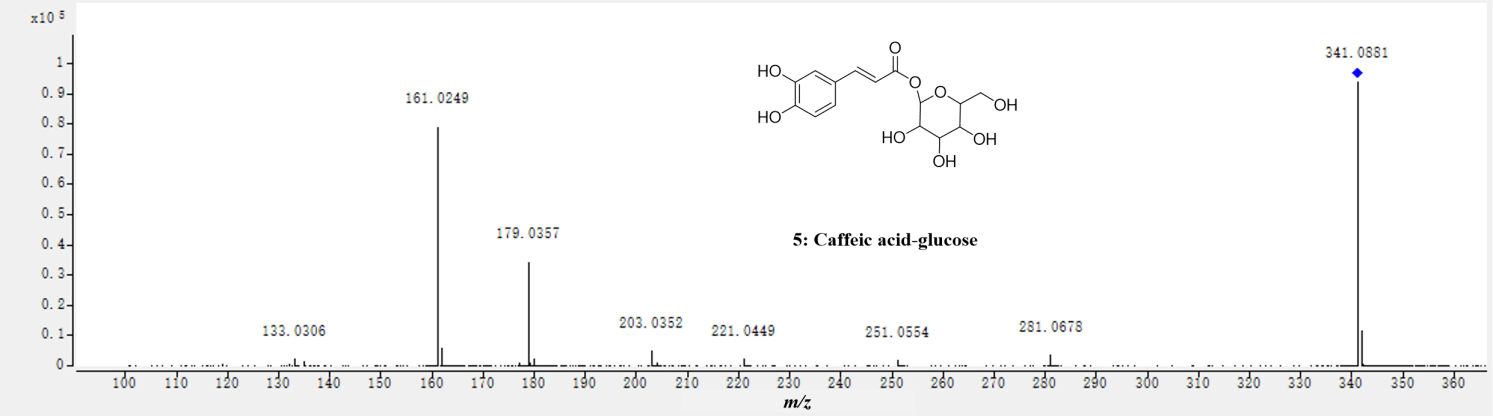


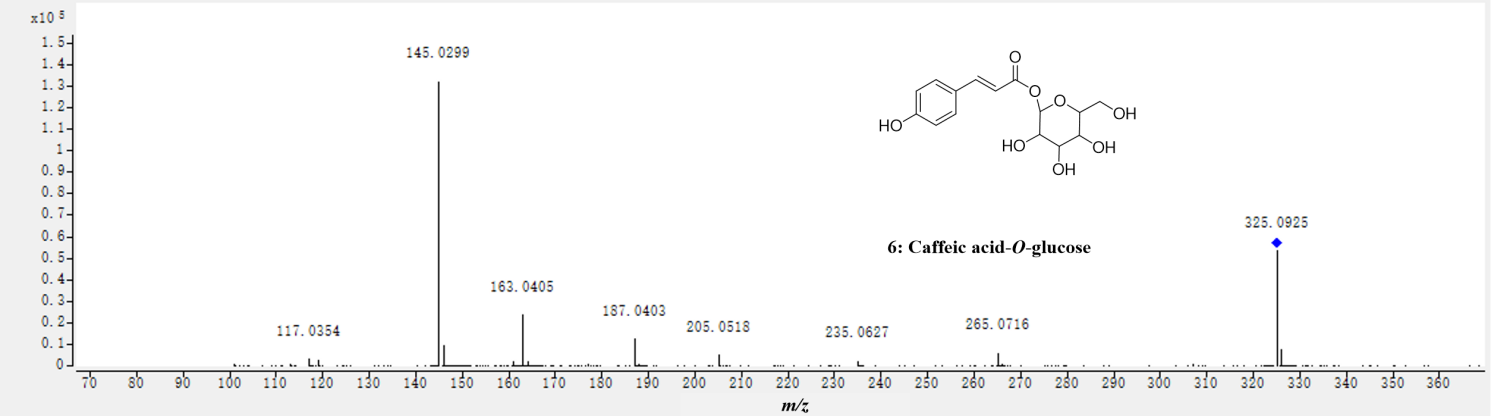


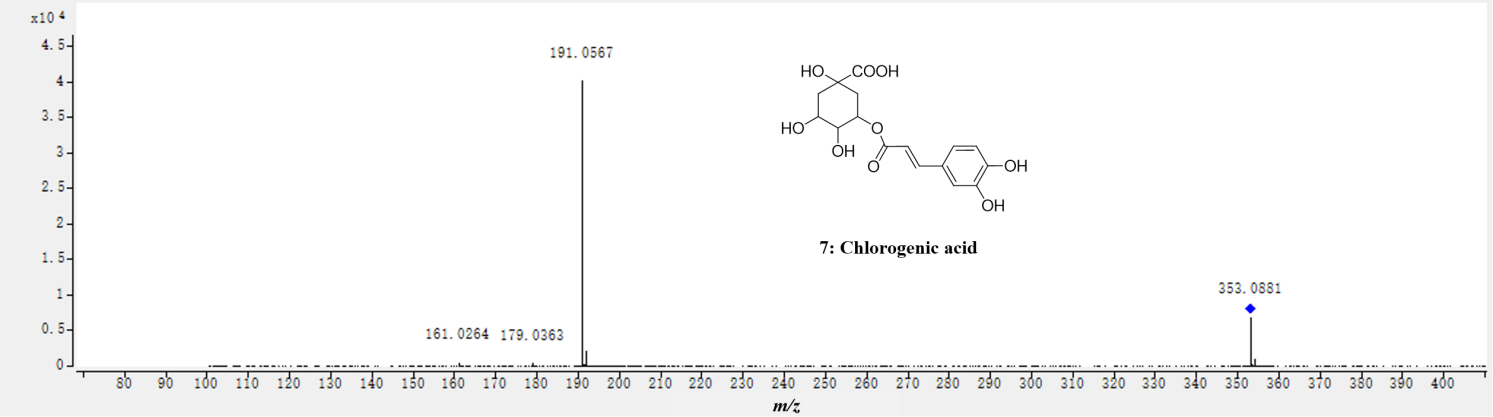


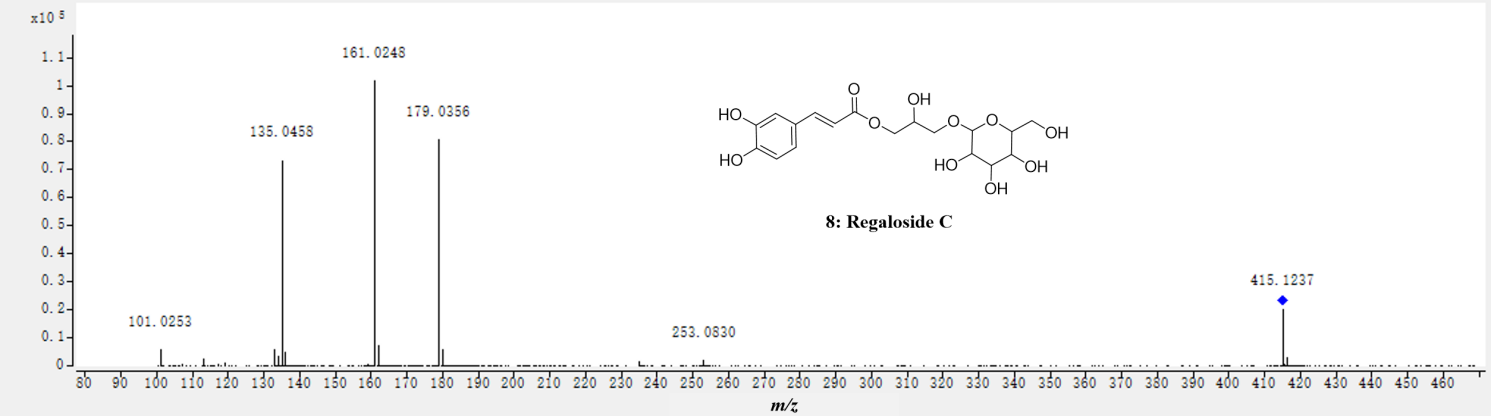


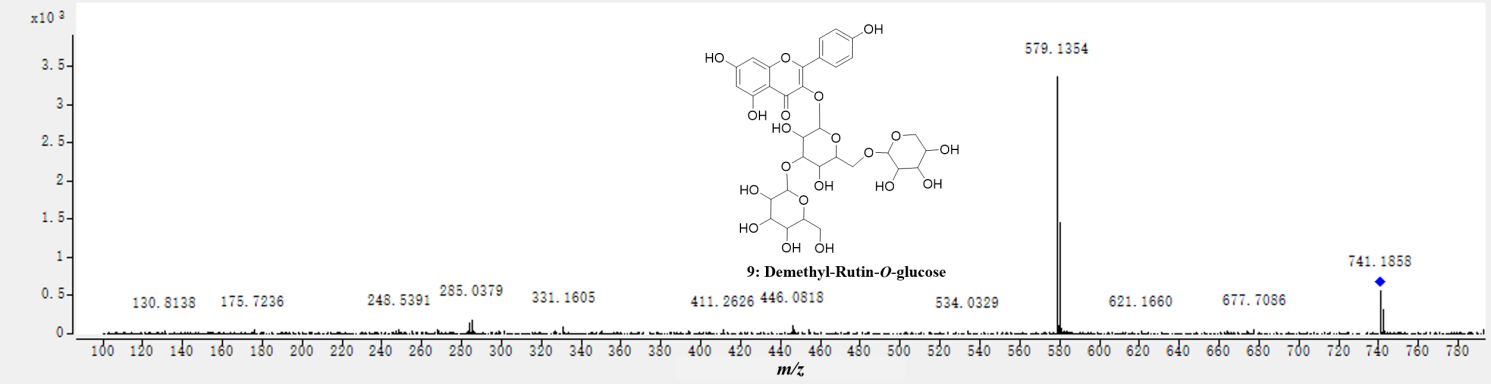


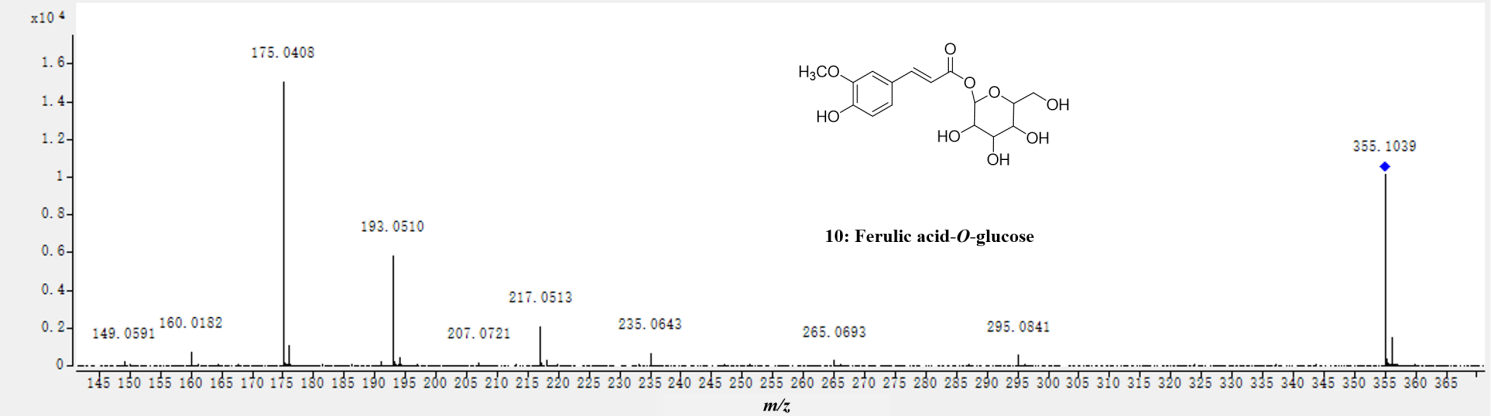


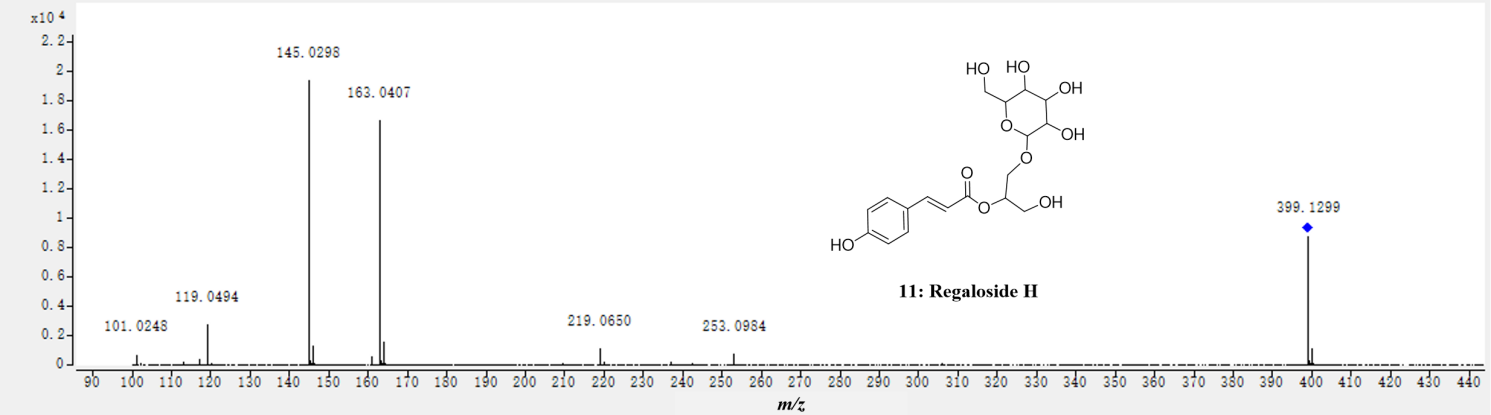


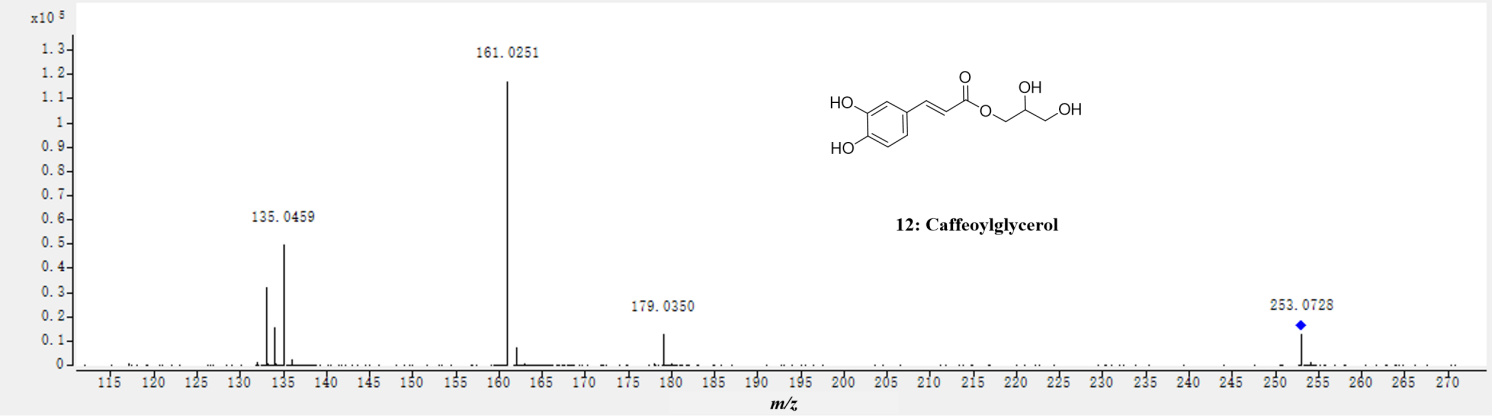


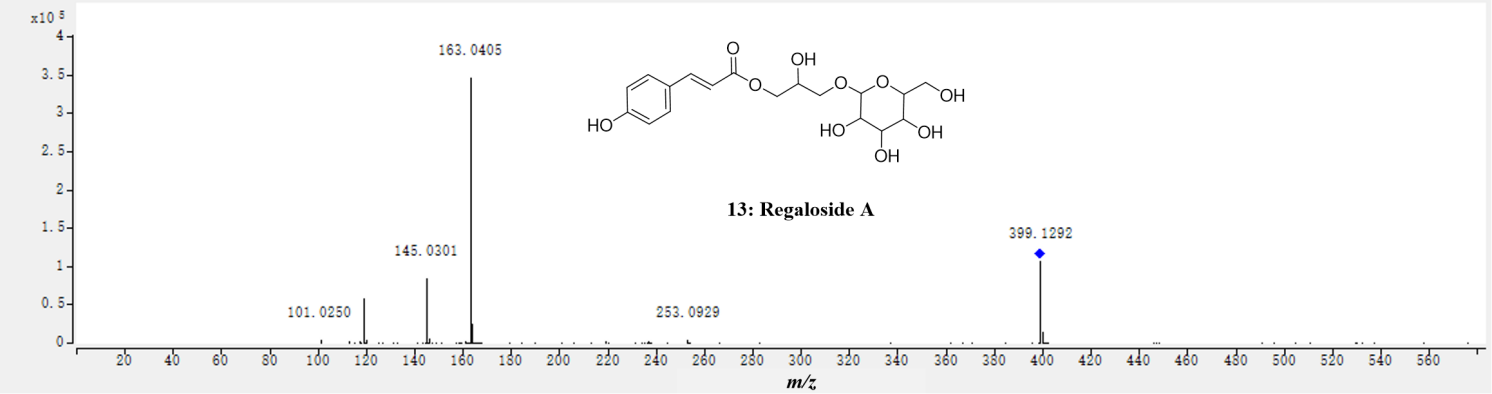


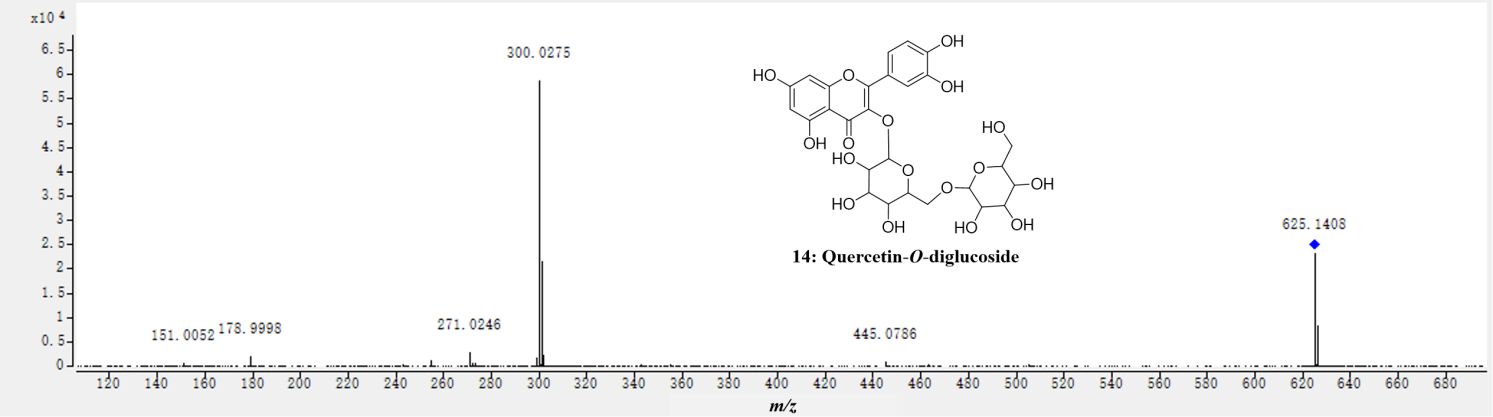


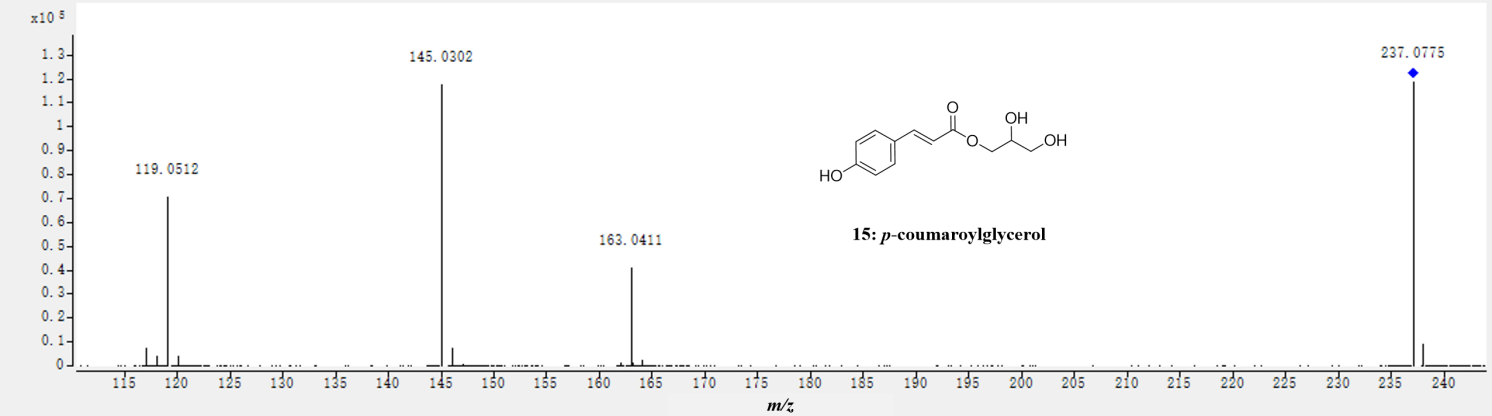


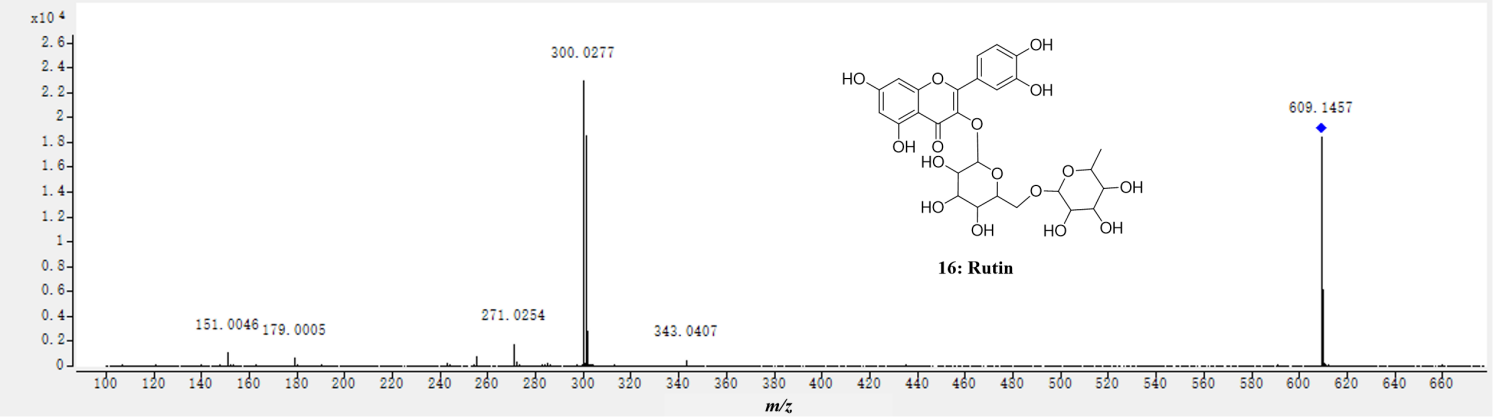


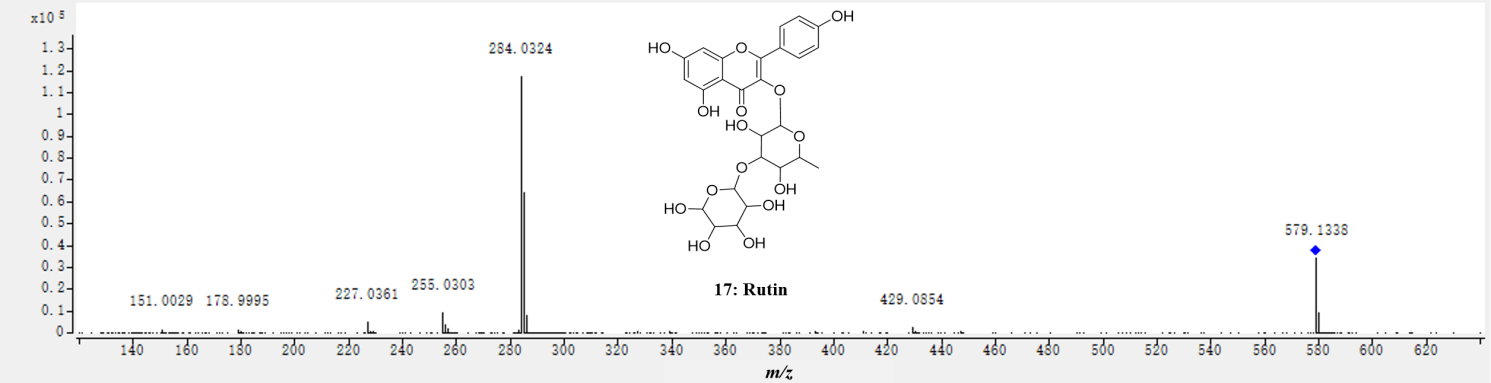


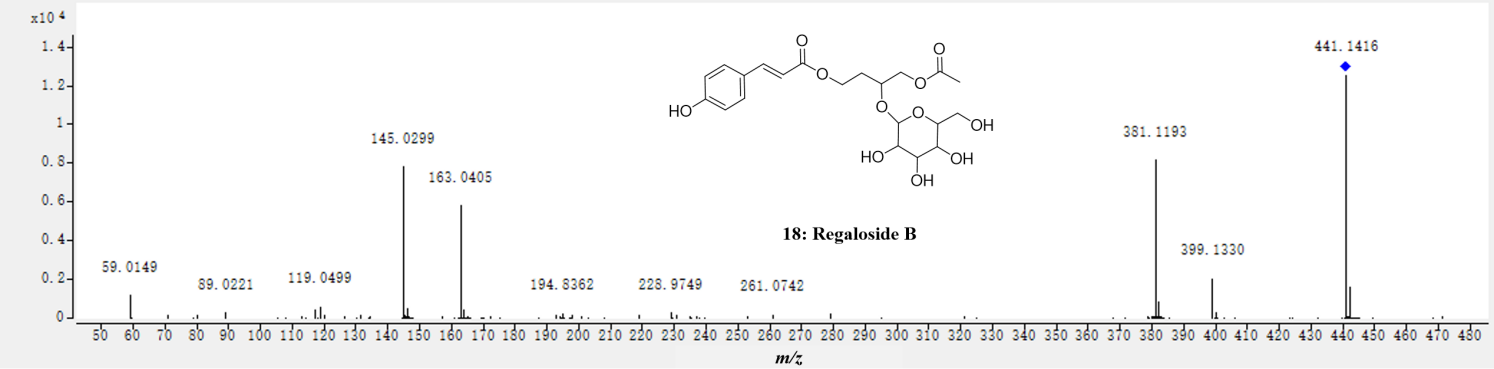


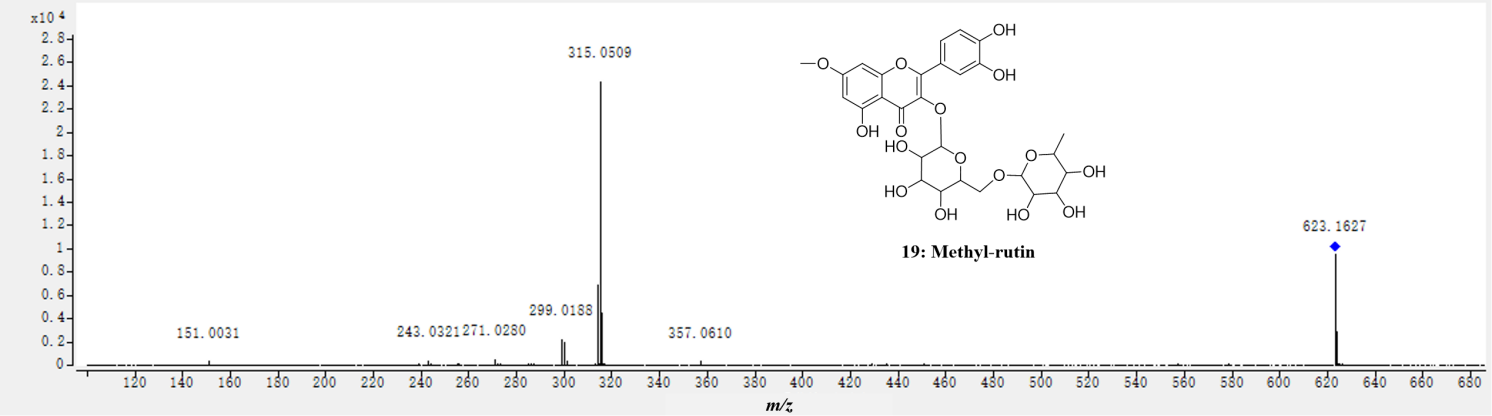


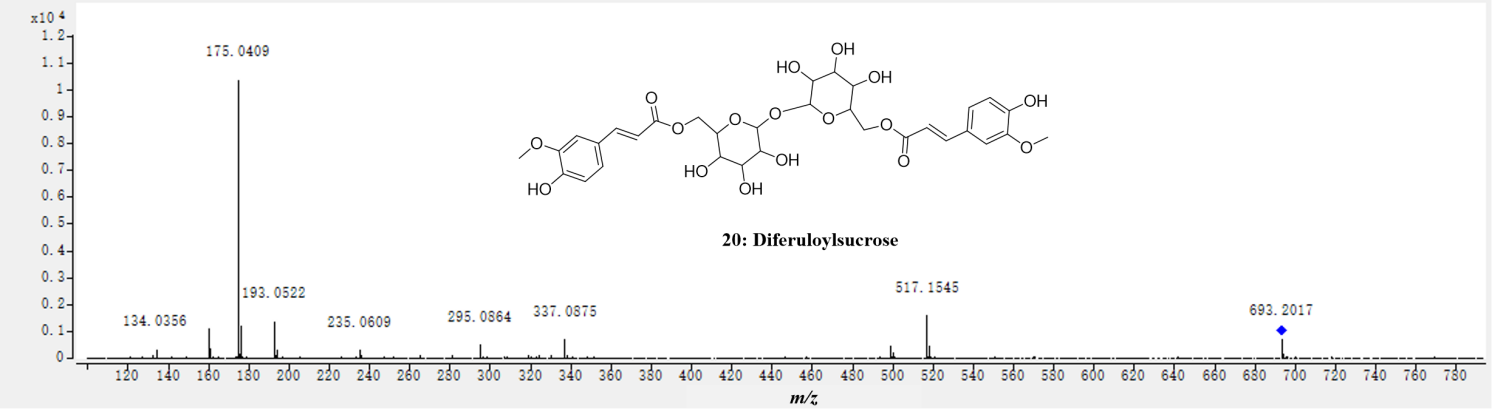


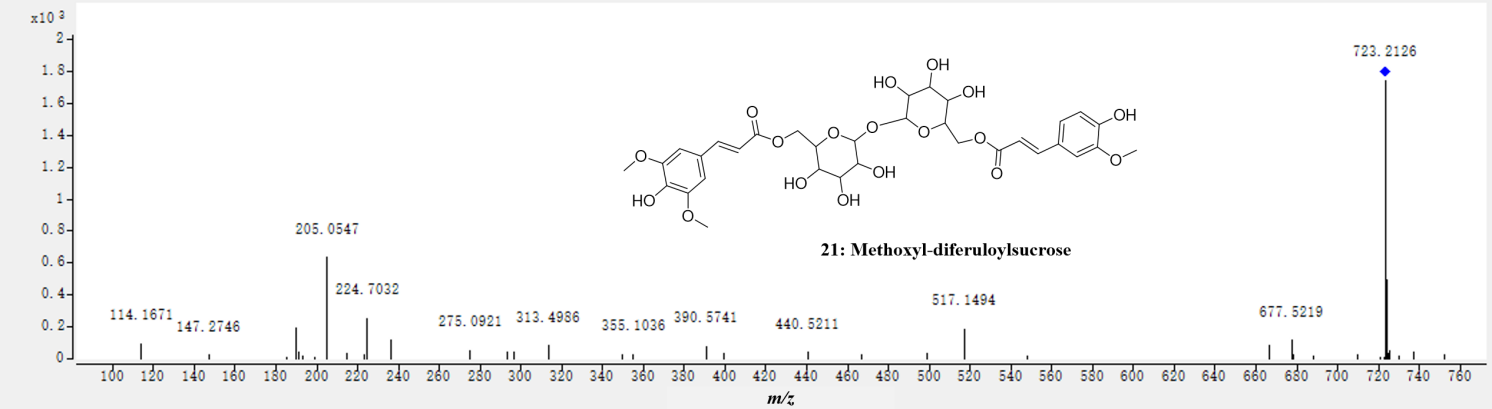


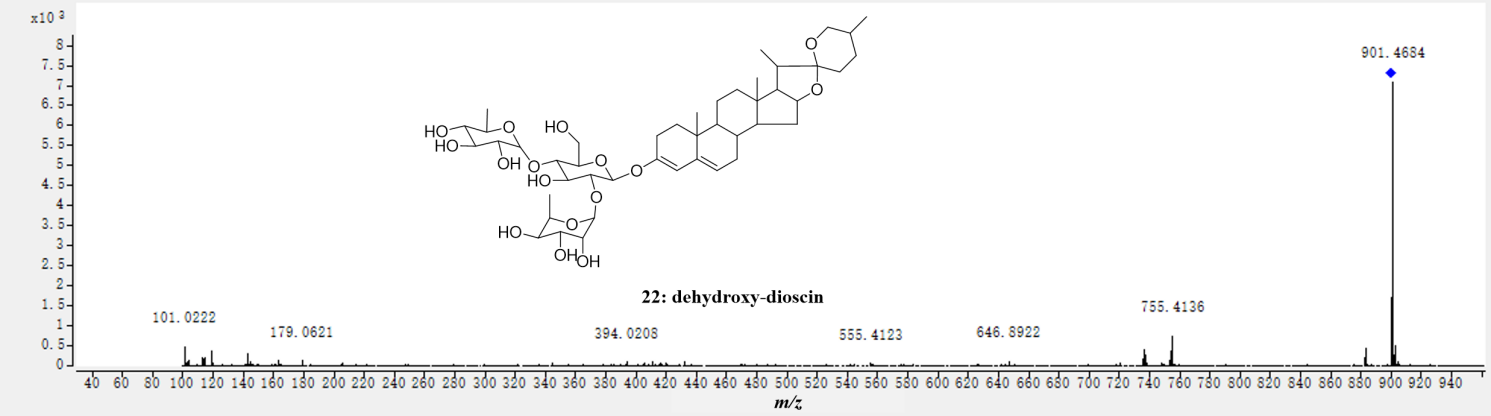


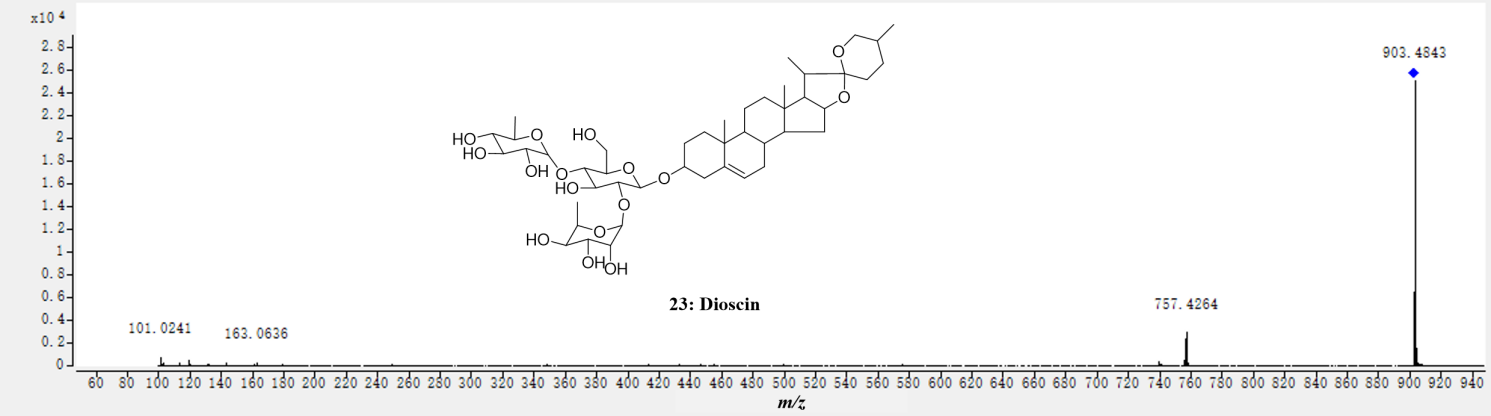


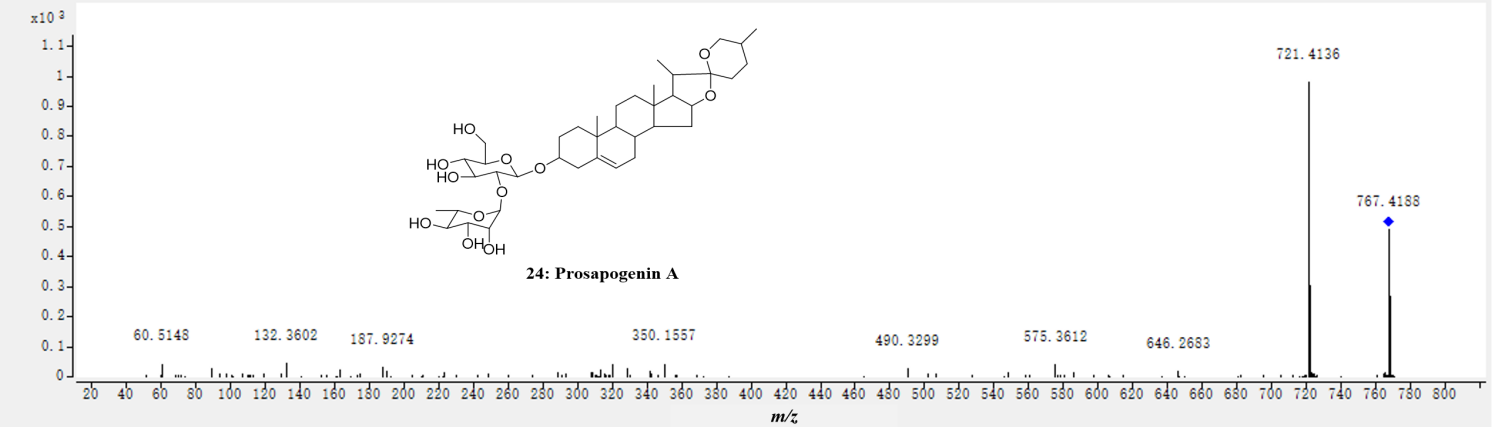


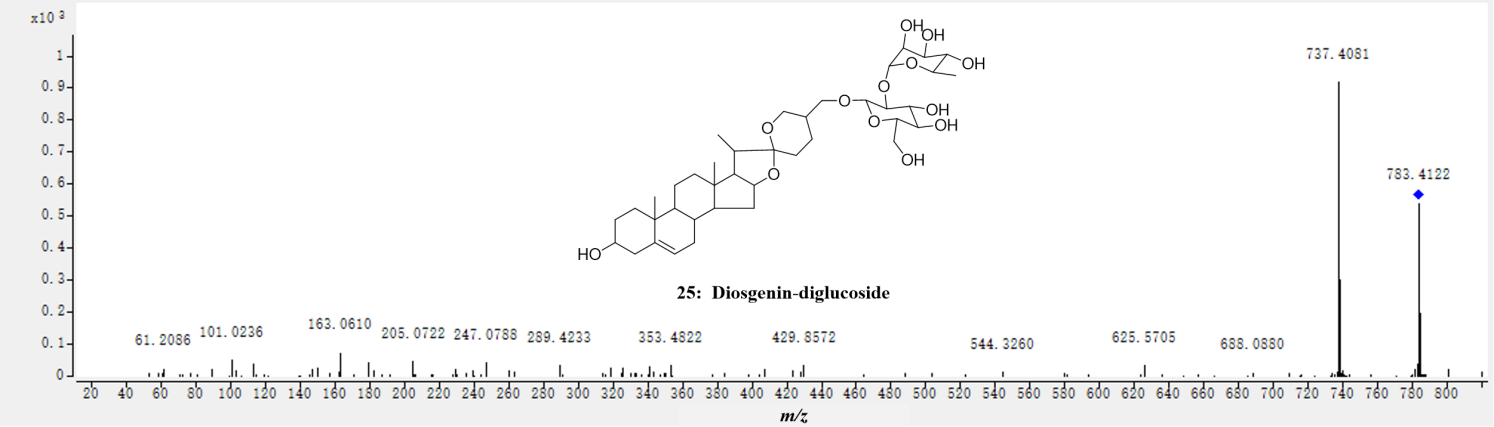


Fig.S2 Molecular docking of acarbose with C-terminal subunit of human maltase-glucoamylase. Overall structure of acarbose with C-terminal subunit of human maltase-glucoamylase (A ). The 3D (B) and 2D images (C) of acarbose with the C-terminal subunit of human maltase-glucoamylase. The 3D images (D) of hydrogen bond acceptor (Green) and donor (Purple). The 3D images (E) of hydrophobic interaction.


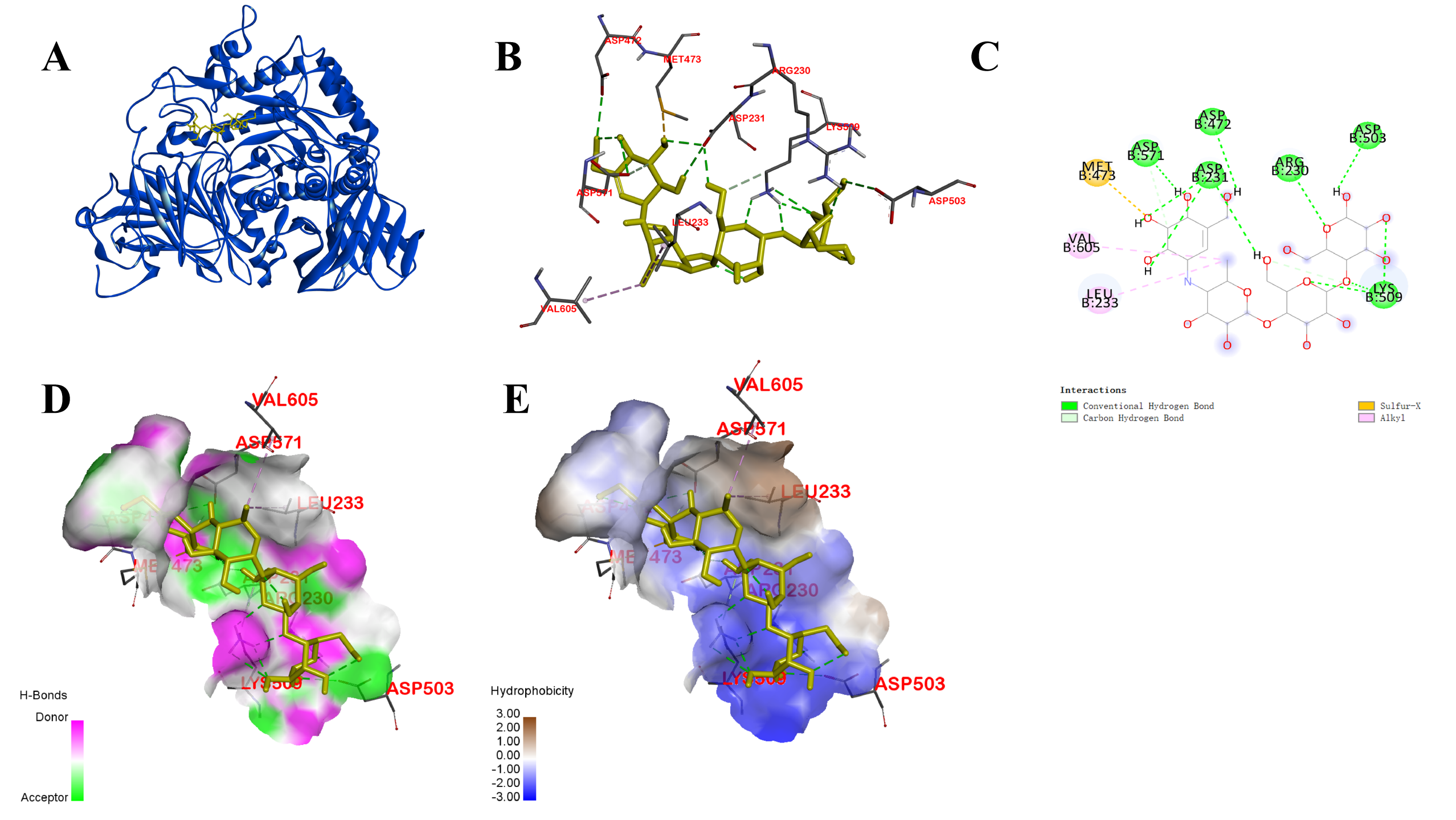


Fig.S3 Molecular docking of acarbose with N-terminal subunit of human maltase-glucoamylase. Overall structure of acarbose with the N-terminal subunit of human maltase-glucoamylase (A ). The 3D (B) and 2D images (C) of acarbose with the N-terminal subunit of human maltase-glucoamylase. The 3D images (D) of hydrogen bond acceptor (Green) and donor (Purple). The 3D images (E) of hydrophobic interaction.


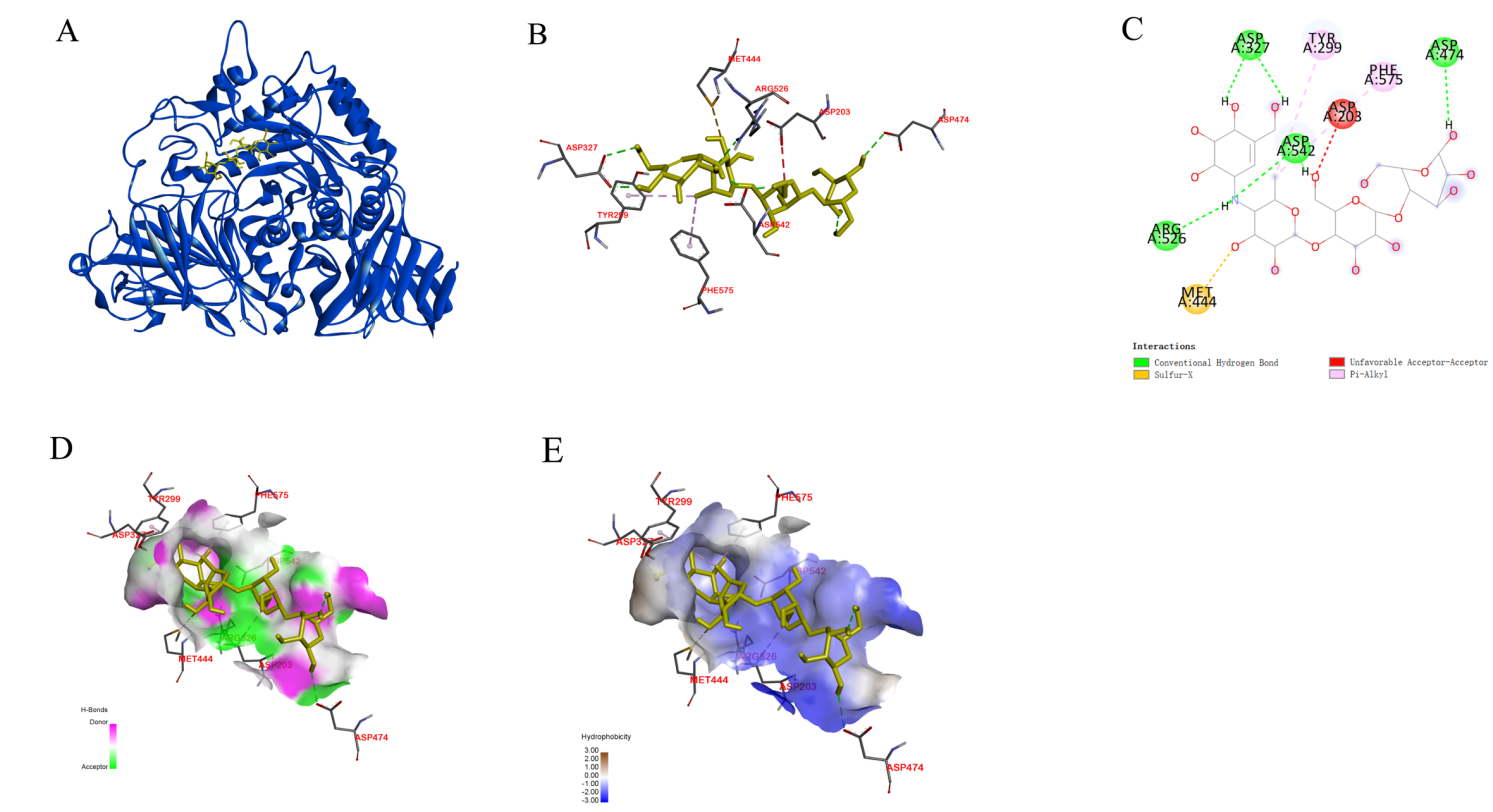


Fig.S4 Molecular docking of regaloside A and B with N-terminal subunit of human maltase-glucoamylase. Overall structure of regaloside A and B with N-terminal subunit of human maltase-glucoamylase (A and F). The 3D (B and G) and 2D images (C and H) of regaloside A and B with the N-terminal subunit of human maltase-glucoamylase. The 3D images (D and I) of hydrogen bond acceptor (Green) and donor (Purple). The 3D images (E and J) of hydrophobic interaction.


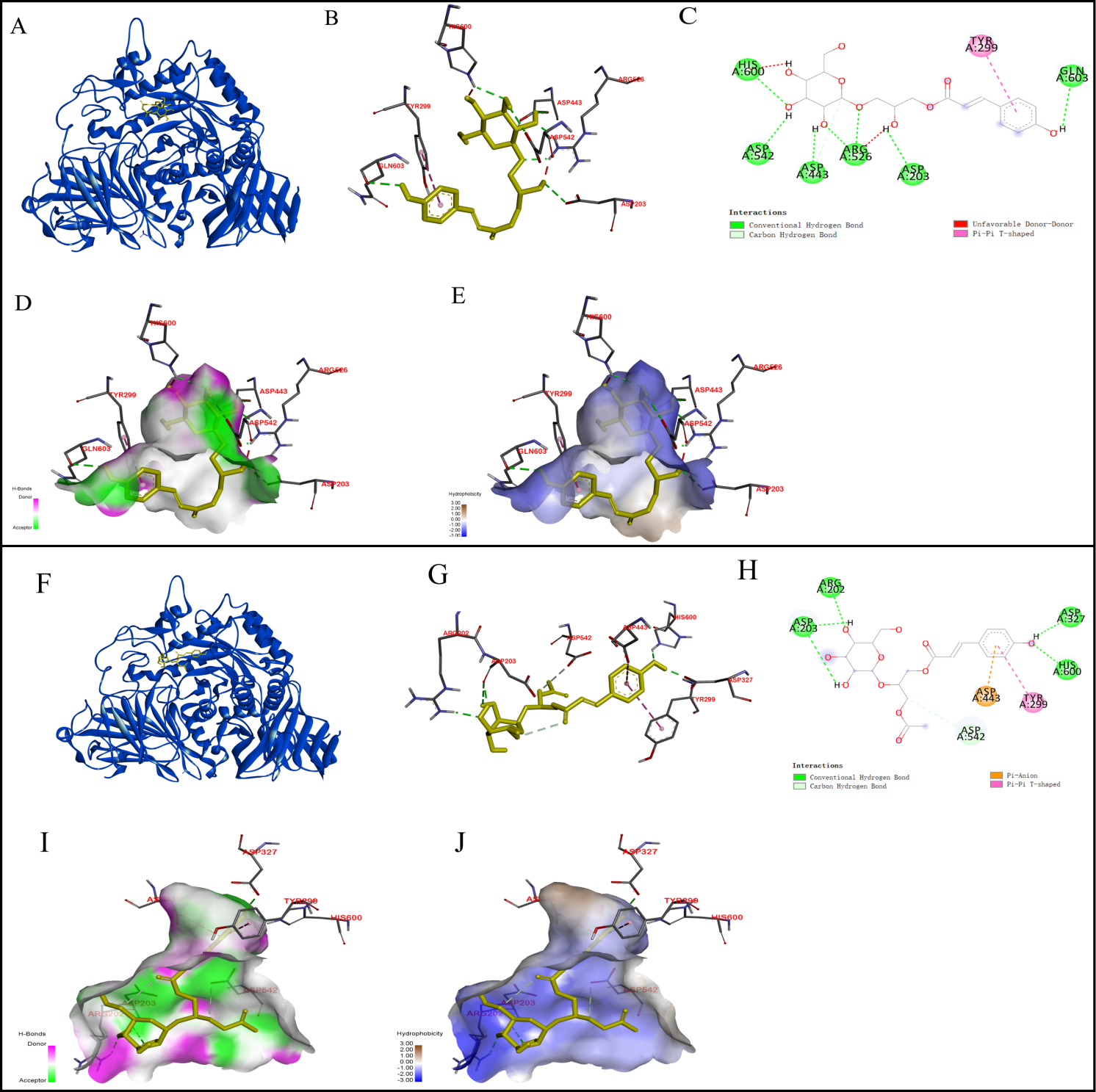


Fig.S5 Molecular docking of acarbose with N-terminal subunit of human sucrase-isomaltase. Overall structure of acarbose with the N-terminal subunit of human sucrase-isomaltase (A ). The 3D (B) and 2D images (C) of acarbose with the N-terminal subunit of human sucrase-isomaltase. The 3D images (D) of hydrogen bond acceptor (Green) and donor (Purple). The 3D images (E) of hydrophobic interaction.


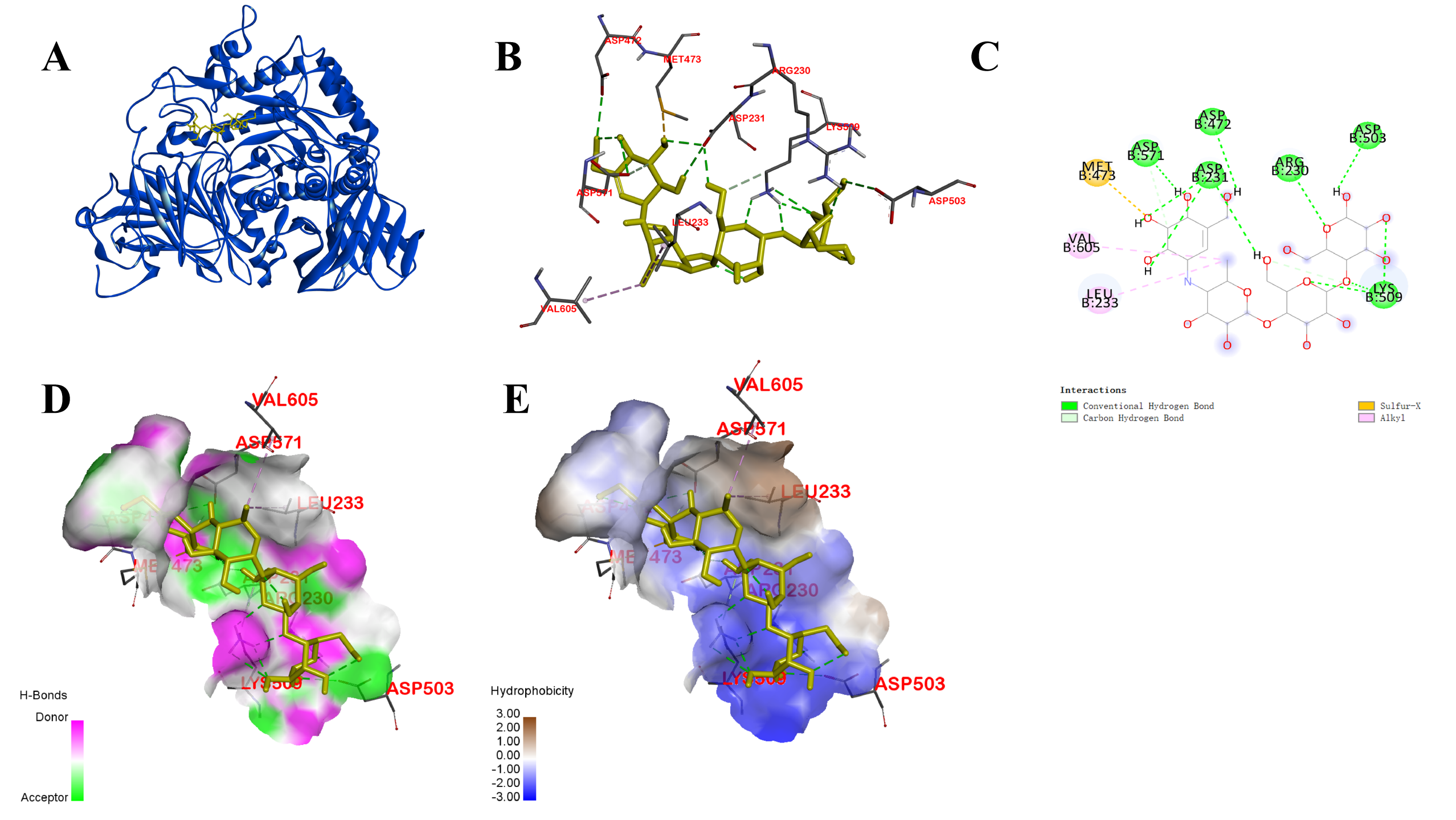


Fig.S6 Molecular docking of regaloside A and B with N-terminal subunit of human sucrase-isomaltase. Overall structure of regaloside A and B with N-terminal subunit of human sucrase-isomaltase (A and F). The 3D (B and G) and 2D images (C and H) of regaloside A and B with the N-terminal subunit of human sucrase-isomaltase. The 3D images (D and I) of hydrogen bond acceptor (Green) and donor (Purple). The 3D images (E and J) of hydrophobic interaction.


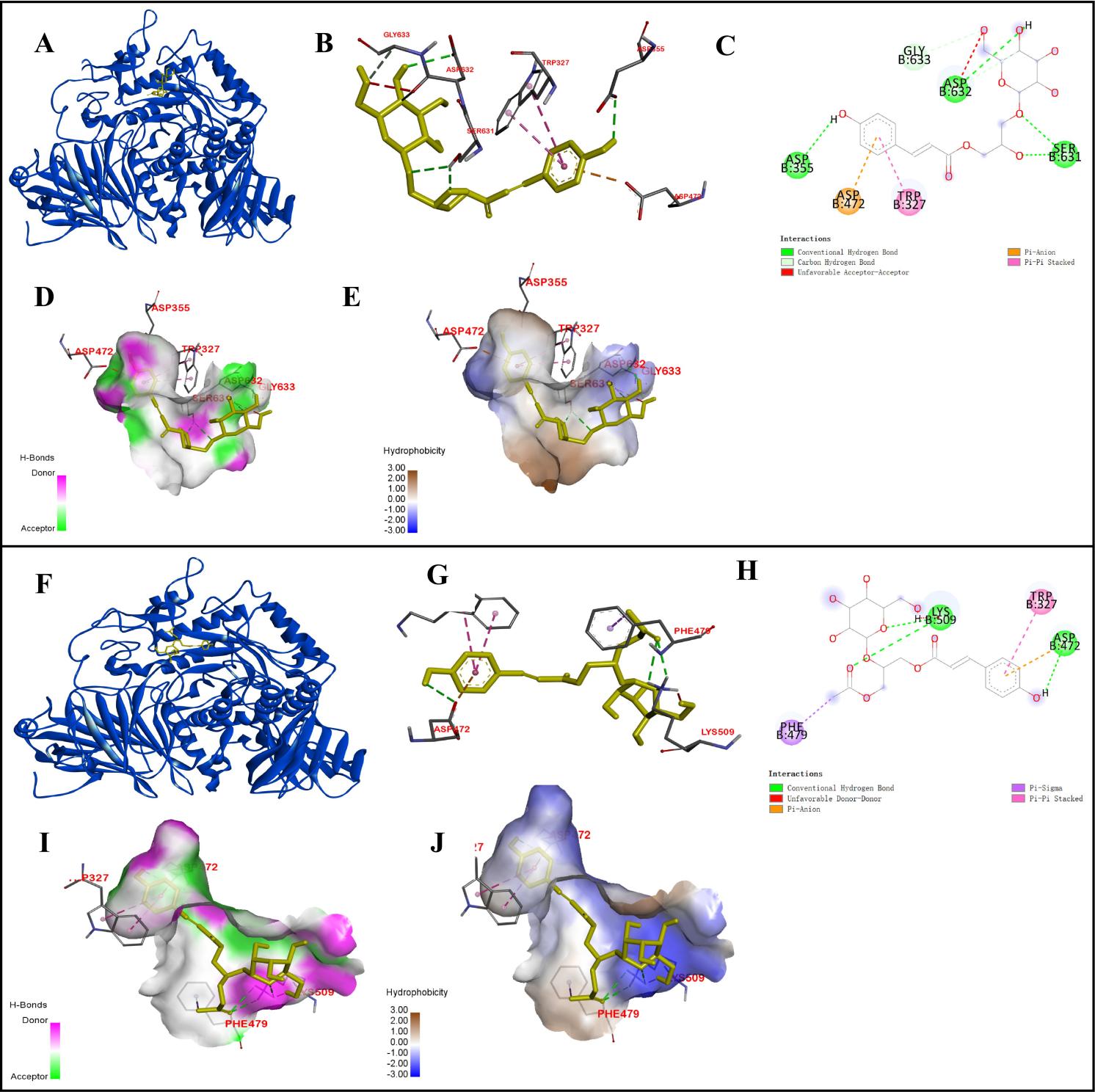

Supplement: Supplementary file 1 [file Supplementaryfile1.docx]
